# Proteomics and cytokine array jointly reveal the role of macrophage proinflammatory shift in liver fibrosis in dairy cows with ketosis

**DOI:** 10.1186/s40104-025-01219-4

**Published:** 2025-07-08

**Authors:** Shiquan Zhu, Moli Li, Yihui Huo, Qiqi Cao, Zhaoju Deng, Kui Li, Yuxin He, Jian Gao, Chuang Xu

**Affiliations:** National Key Laboratory of Veterinary Public Health and Safety, College of Veterinary Medicine, China Agricultural University, 2 Yuanmingyuan West Road, Beijing, 100193 China

**Keywords:** Cytokine network dysregulation, Liver fibrosis, Macrophage, Mitochondrial fission

## Abstract

**Background:**

Changes in macrophage function are crucial contributors to hepatic inflammation and fibrosis. However, the role of macrophages in the development of liver fibrosis in dairy cows with ketosis remains unclear. This study integrated proteomics and cytokine array approach to identify the multifactorial and multicellular interaction effects driving liver fibrosis in dairy cows with ketosis and analyze the mechanism by which the proinflammatory shift in macrophages contributes to liver fibrosis.

**Results:**

Histopathological analysis revealed liver injury, including severe steatosis, infiltration of inflammatory cells, an increase in lipid deposition, and a decrease in glycogen expression in ketotic cows. Moreover, the number of mitochondria considerably increased in hepatocytes. The activation of the dynamin-related protein 1/mitochondrial fission factor (DRP1/MFF) pathway induced excessive mitochondrial fission, and the inhibition of the nuclear factor erythroid 2-related factor 2/heme oxygenase 1 (Nrf2/HO-1) pathway led to the accumulation of intracellular reactive oxygen species (ROS). Proteomic analysis revealed the activation of extracellular matrix (ECM)-related functions and the NF-κB pathway in the liver, whereas cytokine array analysis revealed that the cytokine network was dysregulated. The accumulation of ROS triggered NF-κB nuclear translocation, inducing a proinflammatory shift in macrophages and liver inflammation. M1 polarization of macrophages promotes the release of proinflammatory mediators, which stimulated hepatic stellate cells (HSCs) activation, leading to ECM deposition, ultimately contributing to liver fibrosis.

**Conclusions:**

To summarize, our study revealed the multifactorial and multicellular interaction effects driving liver fibrosis. Our results preliminarily showed that increased mitochondrial fission and inhibition of the Nrf2/HO-1 pathway are key factors in activating macrophages, which can lead to liver fibrosis in dairy cows with ketosis.

**Supplementary Information:**

The online version contains supplementary material available at 10.1186/s40104-025-01219-4.

## Introduction

Ketosis is widely recognized as a significant metabolic disorder in dairy cows, reflecting the animal’s inability to adapt to the energy deficit that occurs during the transition into lactation [1, 2]. During the peripartal period, the onset of lactation increases the energy demands of dairy cows, whereas a simultaneous decrease in feed intake drives them into a state of negative energy balance (NEB) [3]. This NEB triggers fat mobilization, increasing the levels of circulating non-esterified fatty acids (NEFAs). When NEFA accumulation exceeds the liver’s processing capacity, producing large amounts of ketone dominated by β-hydroxybutyric acid (BHBA), leading to the onset of ketosis [4]. Dairy cows with ketosis have an increased risk of developing peripartal health complications, including fatty liver, displaced abomasum, metritis, mastitis, and hypocalcemia [5]. Ketosis also poses significant animal welfare concerns, reduces milk production and quality, and decreases the availability of safe and nutritious food for human consumption [5, 6]. Liver injury is an important characteristic of dairy cows with ketosis and is often accompanied by liver inflammation and oxidative stress, which increase the risk of liver fibrosis [1, 2, 5]. However, the underlying mechanisms that drive the development of liver fibrosis in ketotic cows remain unclear.

The liver is metabolically active in dairy cows with ketosis that operates with high metabolic output at the cost of greater reactive oxygen species (ROS) production [7, 8]. As major intracellular sources and primary targets of ROS, mitochondria are vulnerable to damage during periods of hepatic metabolic burden and stress [9]. In the periparturient period, the liver of dairy cows undergoes tremendous metabolic challenges and oxidative stress, which lead to mitochondrial damage and excessive ROS production [8, 10, 11]. Cows with ketosis exhibit abnormalities in mitochondrial respiratory chain function and dynamics, which contribute to ROS generation and lipid accumulation [10–12]. Mitochondrial dynamics, which include fusion and fission, work in coordination to maintain a healthy mitochondrial population and are necessary for sustaining metabolic homeostasis in response to metabolic disturbances [13]. A key function of mitochondrial fission is the segregation of damaged mitochondrial components to maintain cellular homeostasis [14]. Abnormal mitochondrial fission directly facilitates oxidative stress, further contributing to metabolic disorders and fibrosis in the liver [15]. Overactivation of mitochondrial fission triggers mitochondrial network fragmentation, exacerbates ROS overproduction, and subsequently drives inflammation and the progression of fibrosis [16, 17]. Thus, abnormal mitochondrial fission as a potential factor driving the development of liver metabolic dysfunction and fibrosis in dairy cows with ketosis needs to be further studied.

Macrophages are key regulators of hepatic inflammation and constitute two major subsets: liver-resident macrophages (Kupffer cells) and recruited monocyte-derived macrophages from peripheral blood [18]. The dynamics of liver macrophage turnover and function experience changes during the development of metabolic liver disease [19]. According to the classical M1/M2 theory, activated macrophages are categorized into M1 and M2 phenotypes. In mice fed a high-fat diet, infiltration of macrophages with a predominant M1 phenotype leads to the production of proinflammatory cytokines, resulting in severe inflammation-induced hepatic injury [20]. Pharmacological changes in macrophage polarization toward the alternatively activated M2 phenotype are associated with attenuation of hepatic injury [21]. Macrophage infiltration into the liver is a hallmark and a cause of hepatic inflammatory injury [19]. Excessive ROS production triggers a proinflammatory shift in the macrophage phenotype, thereby facilitating liver inflammation [22–24]. We hypothesized that ROS can trigger a proinflammatory shift in macrophages, which in turn drives the development of liver fibrosis in dairy cows with ketosis. To test this hypothesis, we integrated proteomics and cytokine array analysis to elucidate the molecular mechanisms underlying macrophage polarization and its contribution to liver fibrosis in dairy cows with ketosis.

## Materials and methods

### Animal ethics statement

All experimental procedures involving animals were approved by the Animal Welfare Committee of China Agricultural University (permit No. AW72604202-2-1).

### Animals and sample collection

This study was conducted using Holstein dairy cows from a large-scale intensive farm in Hebei Province, China. All cows were fed a total mixed ration thrice daily at 0700, 1300, and 2100 h, allowing ad libitum intake. Fresh water was supplied continuously. The basal diet formulation is provided in Table S1.

Blood BHBA levels were measured in 240 Holstein dairy cows with a similar number of lactations (median = 3, range = 2–4) and days in milk (median = 11 d, range = 5–14) using an electronic BHBA hand-held meter (Precision Xtra, Abbott). As demonstrated in a previous study, the hand-held meter, when measuring whole blood at a threshold of 1.4 mmol/L, exhibited a sensitivity of 100% (69%–100%), specificity of 100% (94%–100%), positive predictive value of 100% (69%–100%), and negative predictive value of 100% (98%–100%) for detecting ketosis in dairy cows [25]. Based on clinical symptoms and BHBA levels, 30 dairy cows were selected and categorized into two groups: normal (CON group; *n* = 15; serum BHBA concentrations ≤ 0.6 mmol/L) and clinical ketosis (KET group; *n* = 15; serum BHBA concentrations ≥ 3.0 mmol/L) groups. Blood samples were collected from these 30 dairy cows via coccygeal venipuncture before the morning feeding. The serum was separated by centrifugation at 3,500 × *g* at 4 °C for 15 min. Liver biopsies were performed on a final cohort of 10 cows (five clinically ketotic cows and five control cows). The characteristics, including body weight (BW), dry matter intake (DMI), and milk production, of the selected normal cows and cows with clinical ketosis are listed in Table S2. Consistent with previous research, dairy cows with ketosis presented lower milk production and DMI [7].

Liver tissue samples were obtained by shaving the intercostal space, disinfecting it with an iodine scrub and 75% alcohol, and administering local anesthesia with 2% lidocaine hydrochloride via subcutaneous injection. A 3-mm incision was made in the skin with a scalpel, and a liver puncture needle was inserted through the incision and advanced into the liver. About 1.5–2 g (wet weight) of liver tissue was obtained as described previously [26, 27]. The liver tissue was carefully washed with sterile isotonic saline solution to remove blood contamination, immediately cut into small pieces using a scalpel, and fixed in 4% paraformaldehyde, optimal cutting temperature (OCT) compound, or 2.5% glutaraldehyde phosphate buffer, or frozen in liquid nitrogen and stored at −80 °C until analysis.

### Histopathological observation

After the liver tissue samples were fixed with 4% paraformaldehyde, they were dehydrated in ethanol and embedded in paraffin. A 5-μm section was obtained and stained with hematoxylin and eosin (H&E). Liver lipid accumulation was observed via Oil Red O staining. After fixing for 24 h in 4% paraformaldehyde, the liver tissues were dehydrated in a gradient concentration of sucrose solution. The tissues were embedded in OCT compound, frozen quickly, cut into 10-μm slices, and stained with Oil Red O solution. The liver glycogen content was observed via periodic acid-Schiff (PAS) staining. The prepared paraffin sections were dewaxed with xylene and rehydrated using an ethanol gradient. Then, the slices were treated with periodic acid for 15 min, Schiff’s reagent for 30 min, and hematoxylin for 30 s. The glycogen molecules were stained purplish red, and the nuclei were stained blue. For fibrosis staining, the tissue sections underwent Masson and Sirius Red staining. The sections were observed under a light microscope (Olympus, Japan) or a polarized light microscope (Nikon Eclipse Ci, Japan). ImageJ (version 1.53) was used to quantitatively assess the percentage of the vacuolar area, Oil Red O-positive area, glycogen-positive area, and fibrosis area in liver tissues.

### Serum biochemical parameter analysis

After the blood was centrifuged, 200 µL of serum was collected for biochemical parameter detection. Serum biochemical parameters, including NEFA (#09595066001), glucose (GLU, #73288001), alanine aminotransferase (ALT, #72938001), aspartate aminotransferase (AST, #71671801), gamma-glutamyl transferase (GGT, #74415401), total protein (TP, #72256101), and albumin (ALB, #72570901), were measured using commercially available kits (Roche Diagnostics, Indianapolis, USA) on an AutoAnalyzer (Roche Cobas 6000, Germany) following the manufacturer’s instructions. Globulin (GLB) was obtained by subtracting ALB from TP.

### Ultrastructural analysis

Fresh liver tissues were cut into small pieces of 1 mm^3^. The samples were quickly immersed in 2.5% glutaraldehyde solution at 4 °C. The samples were subsequently fixed in OsO_4_, dehydrated, and embedded in epoxy resin. Ultrathin 50-nm-thick sections were cut and stained with uranyl acetate and lead citrate. Images were captured via transmission electron microscopy (TEM) (Hitachi H-7800, Japan). Mitochondrial morphological parameters (area, diameter, and perimeter) were measured using the ImageJ software (version 1.53).

### Determination of oxidative stress indicators

About 50 mg of liver tissue was used to determine the activities of total superoxide dismutase (T-SOD; A001-1), glutathione peroxidase (GSH-Px; A005-1), and catalase (CAT; A007-1) and the levels of malondialdehyde (MDA; A003-1) and total antioxidant capacity (T-AOC; A015-2) using commercially available kits (Nangjing Jiancheng Bioengineering Institute, Nangjing, China), following the manufacturer’s protocol.

### Enzyme-linked immunosorbent assay (ELISA) detection of proinflammatory cytokines and 8-hydroxydeoxyguanosine (8-OHdG)

About 40 mg of liver tissue was weighed for homogenization, and the samples were diluted 2–3-fold before analysis. The concentrations of monocyte chemoattractant protein-1 (MCP-1; SEA087BO), interleukin 1 beta (IL-1β; SEA563Bo), interleukin 6 (IL-6; SEA079Bo), and an oxidative damage marker (8-OHdG; CEA660Ge) in the liver were detected using commercial ELISA kits (Uscn Life Science Inc., Wuhan, China) following the manufacturer’s instructions. Each sample was analyzed in triplicate. The detection limits were as follows: 8-OHdG (74.07 pg/mL–6,000 pg/mL), IL-1β (15.6–1,000 pg/mL), IL-6 (7.8 pg/mL–500 pg/mL), and MCP-1 (15.6 pg/mL–1,000 pg/mL).

### Detection of hepatic ROS production

To assess the levels of ROS in liver tissues, a dihydroethidium (DHE) fluorescence probe was used. The 10-μm-thick frozen slices were incubated with DHE (10 mmol/L) at 37 °C for 30 min, after which the cell nuclei were stained with DAPI. The fluorescence of DHE was visualized via fluorescence microscopy (Olympus, Japan). The fluorescence intensity of DHE-stained samples was quantitatively analyzed using the ImageJ software (version 1.53).

### Terminal dUTP nick-end labeling (TUNEL) and phosphorylated H2A histone family member X (γH2AX) double-staining assays for evaluating apoptosis and DNA oxidative damage

Hepatic apoptosis and DNA oxidative damage were evaluated by conducting TUNEL and γH2AX double-staining assays. Briefly, the paraffin-embedded tissue sections were dewaxed in xylene, repaired with protease K, permeabilized with Triton X-100, and labeled with TdT incubation buffer. Next, the sections were incubated with γH2AX primary antibodies for 12 h at 4 °C and Cy3-conjugated goat anti-rabbit IgG secondary antibodies for 50 min at 25 °C. DAPI was used to stain the nuclei. The sections were observed under a fluorescence microscope (Olympus, Tokyo, Japan). The rate of TUNEL-positive nuclei, γH2AX-positive nuclei and double-positive nuclei of TUNEL and γH2AX were determined for each group.

### Quantitative reverse transcription polymerase chain reaction (qRT-PCR)

For extracting total RNA, 20 mg of liver tissue was weighed. RNA extraction and real-time PCR were performed as follows. Briefly, total RNA was isolated from liver tissues using the reformative technique with TRIzol reagent (Invitrogen, USA). Then, cDNA was generated using a reverse transcription kit (Takara, Japan). Quantitative PCR assays were conducted using the SYBR® Premix Ex Taq™ kit (Takara, Japan). Target gene expression was quantified using the 2^–△△Ct^ method and normalized to the expression of GAPDH. The PCR primer sequences are shown in Table S3.

### Immunofluorescence staining

For fluorescent single-label staining, the liver tissue sections were dewaxed and placed in EDTA buffer for antigen retrieval by heating in a microwave oven for 15 min. After washing, the sections were blocked with BSA for 30 min. Subsequently, the samples were incubated with primary antibodies overnight at 4 °C and secondary antibodies for 50 min at 25 °C. The antibodies were diluted in PBS (G4202, Servicebio). DAPI was then used to counterstain the nuclei. Images were acquired via fluorescence microscopy and analyzed using the ImageJ software (version 1.53).

For fluorescent homologous double-labeling staining, the paraffin sections were dewaxed in water. Then, antigen retrieval and enclosed endogenous peroxidase were performed. After blocking with 3% BSA for 30 min, the primary antibodies were added to the slides for incubation at 4 °C overnight. After washing, the slides were incubated with a polymer horseradish peroxidase (HRP)-conjugated antibody specific to rabbits. After rinsing, the corresponding TSA dye was added to each slide and incubated at room temperature. Subsequently, antigen retrieval was performed on the stained slides to allow staining for the next target protein. The antibodies, dilution factors, sources, and other information are presented in Table S4. Nuclei were counterstained with DAPI. All images were captured using a fluorescence microscope and analyzed using the ImageJ software (version 1.53).

### Proteomic analysis

Three liver samples from each group were used in this study, and 20 mg of liver tissue was weighed for protein extraction. Briefly, liver tissue was transferred to lysis buffer (1% SDS, 8 mol/L urea, and 1 mg/mL protease inhibitor cocktail), and the resulting supernatant was collected after homogenization and centrifugation at 14,000 × *g* and 4 °C for 20 min. The total protein in the supernatant was quantified using a BCA protein assay kit (P0010, Beyotime, Shanghai, China) following the manufacturer’s instructions. Next, high-pH reversed-phase separation and database searches were conducted. The mass spectrometer was used under data-independent acquisition (DIA) mode and automatically switched between MS and MS/MS modes. The raw DIA data were processed and analyzed using Spectronaut X (Biognosys AG, Switzerland) with default parameters. All selected precursors passing through the filters were used for quantification. The average of the top 3 filtered peptides that passed the 1% *Q*-value cutoff were used to calculate the major group quantities. Proteins with fold changes > 1.2 or < 0.83 and a significance level of *P* < 0.05 were considered to be differentially expressed. Differentially expressed proteins (DEPs) were further annotated against the KEGG database to determine their functions.

### Western blotting analysis

In total, 40 mg of liver tissue was weighed for total protein extraction. The total protein of the liver tissue was extracted with RIPA lysis buffer and centrifuged at 13,000 × *g* for 10 min. The protein concentrations were determined using a BCA protein kit (Abbkine, China). Next, the protein concentrations of the supernatants were determined and normalized to a concentration equal to that of the protein. The targeted proteins were separated via 10% SDS-PAGE and subsequently transferred to a polyvinylidene membrane. After blocking with 5% nonfat milk, specific antibodies, including inhibitors of nuclear factor kappa-B kinase subunit beta (IKKβ), nuclear factor kappa-B (NF-κB) P50 subunit (P50), phospho-NF-κB P65 (p-P65), NF-κB P65 (P65), CD86, inducible nitric oxide synthase (iNOS), CD163, and arginase 1 (ARG1), were used to detect target proteins at an appropriate final concentration following the manufacturer’s instructions. Later, the membrane was rinsed and incubated with secondary antibodies. Finally, the protein bands on the membranes were detected via enhanced chemiluminescence. The antibodies used for Western blotting are shown in Table S5.

### Cytokine array analysis

In total, 30 mg of liver tissue was weighed for cytokine array analysis. The hepatic cytokine profile was quantitatively analyzed using a Multiplex Quantibody Bovine Cytokine Array Q30 kit (RayBiotech, Inc., Guangzhou, China) as described by Smith et al. [28]. The bovine cytokine array was combined with three nonoverlapping arrays to measure 30 bovine cytokines, including cytokines, chemokines, inflammatory mediators, and growth factors, quantitatively. After the liver tissues were lysed, the protein concentrations were measured, and a uniform concentration of 500 µg/mL was used for detection following the manufacturer’s instructions. The differentially expressed cytokines (DECs) were defined as those with value less than 0.05 and fold changes greater than 1.2 or less than 0.83. The GO and KEGG databases were used to perform gene annotation and pathway analysis.

### Analysis strategy for hepatic macrophage activation

Proteomic analysis was performed to identify the significantly enriched pathways associated with macrophage activation. The activation state was further assessed by measuring the expression of inflammatory markers and chemokines through cytokine array analysis. We subsequently validated hepatic macrophage activation by evaluating M1 markers (CD86 and iNOS) and M2 markers (CD163 and ARG1) via immunofluorescence staining, qPCR, and Western blotting. Finally, correlation analysis was conducted to determine the relationships between macrophage activation markers and liver damage.

### Statistical analysis

All data were analyzed using the Prism software (version 10.1.1), and the homogeneity of variance was tested before the analyses. The differences between the groups were determined by conducting two-tailed unpaired Student’s *t*-tests. The data are presented as the mean ± SEM. The differences were considered to be statistically significant at *P* < 0.05. Spearman’s or Mantel’s correlation was performed to analyze the correlation between the macrophage markers and liver damage features. *Q*-values were obtained by adjusting the *P* value using the false discovery rate method. The absolute values of correlation coefficients > 0.5 and *Q* < 0.05 were considered to be significant.

## Results

### Liver histopathological abnormalities and dysfunction in dairy cows with ketosis

**Fig. 1:**
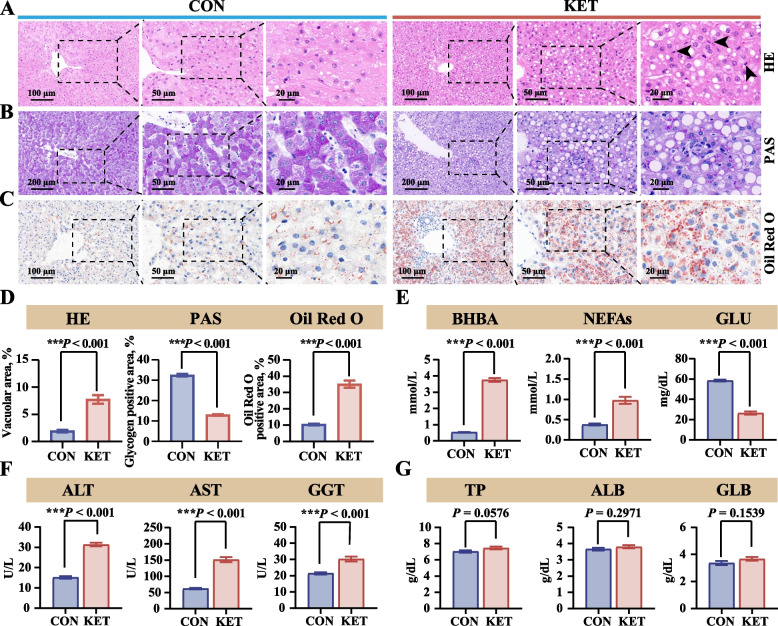
Liver histopathological abnormalities and dysfunction in dairy cows with ketosis. **A–C** Representative images of H&E, PAS, and Oil Red O staining of liver tissues. **D** Quantitative analysis of the percentages of the hepatic vacuolar area, glycogen-positive area, and Oil Red O-positive area of H&E, PAS, and Oil Red O staining, respectively (*n* = 5). **E** Serum levels of BHBA, NEFAs, and GLU (*n* = 15). **F** Liver enzyme activities of ALT, AST, and GGT in the serum (*n* = 15). **G** Serum protein concentrations of TP, ALB, and GLB (*n* = 15). The arrowhead in the HE staining image represents the infiltration of inflammatory cells. The values are expressed as the mean ± SEM. Statistical analysis was performed by conducting unpaired Student’s *t*-tests (two-tailed); ^*^P < 0.05, ^**^P < 0.01, and ^***^P < 0.001

As shown in Fig. 1, H&E, Oil Red O, and PAS staining revealed marked pathological changes in the KET group, including severe hepatic steatosis, increased infiltration of inflammatory cells, reduced glycogen expression, and increased lipid deposition (Fig. 1A–C). Compared to those in the CON group, the percentages of the vacuolar area, glycogen-positive area, and Oil Red O-positive area in the KET group also revealed severe hepatic steatosis, decreased glycogen expression, and increased lipid deposition (Fig. 1D). The levels of serum BHBA and NEFAs were significantly greater, whereas GLU levels were lower in the KET cows than in the CON cows (Fig. 1E). Several key hepatic function-related serum biochemical indicators were detected to assess liver damage. AST, ALT, and GGT activities collectively reflect liver function. The results revealed that the activities of serum liver function-associated enzymes (ALT, AST, and GGT) were significantly greater in the KET cows than in the CON cows (Fig. 1F). The serum protein concentrations of TP, ALB, and GLB were not significantly different between the groups (Fig. 1G).

### Excessive mitochondrial fission and inhibition of the Nrf2/HO-1 pathway contribute to ROS accumulation in the hepatocytes of ketotic cows

**Fig. 2:**
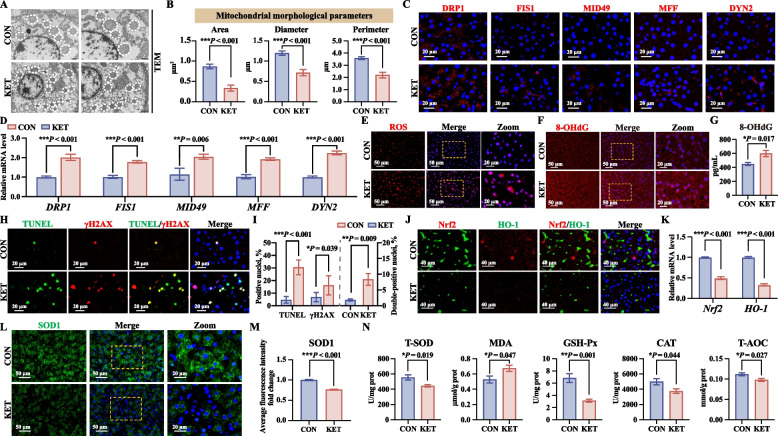
Excessive mitochondrial fission and inhibition of the Nrf2/HO-1 pathway contribute to hepatocellular ROS accumulation. **A** Transmission electron microscopy (TEM) images of mitochondria in hepatocytes. **B** Mitochondrial morphological parameters are shown as area, diameter, and perimeter (*n* = 5). **C** Immunofluorescence images of Dynamin-related protein 1 (DRP1), Mitochondrial fission 1 protein (FIS1), Mitochondrial dynamics protein 49 (MID49), Mitochondrial fission factor (MFF), and Dynamin-2 (DYN2) (*n* = 5). **D** qRT-PCR analysis was conducted to determine the mRNA expression levels of *DRP1*, *FIS1*, *MID49, MFF*, and *DYN2* (*n* = 5). **E** Intracellular ROS production (red). **F** and **G** Immunofluorescence images and concentrations of 8-hydroxydeoxyguanosine (8-OHdG). **H** Immunofluorescence double-staining images of terminal dUTP nick-end labeling (TUNEL, green) and phosphorylated H2A histone family member X (γH2AX, red). **I** Quantitative analysis of the percentage of positive nuclei (*n* = 5). **J** Immunofluorescence double-stained images of nuclear factor erythroid 2-related factor 2 (Nrf2, red) and heme oxygenase 1 (HO-1, green) expression. **K** qRT-PCR analysis was conducted to determine the mRNA expression levels of *Nrf2* and *HO-1* (*n* = 5). **L** and **M** Immunofluorescence staining images and average fluorescence intensity fold change of Cu/Zn superoxide dismutase (SOD1). **N** The levels of oxidative stress-related indicators (T-SOD, MDA, GSH-Px, CAT, and T-AOC) in liver tissues (*n* = 5). The values are expressed as the mean ± SEM. Statistical analysis was performed by conducting unpaired Student’s *t*-tests (two-tailed); ^*^P < 0.05, ^**^P < 0.01, and ^***^P < 0.001

Liver ultrastructure was analyzed via TEM, and hepatic mitochondrial morphological parameters (area, diameter, and perimeter) were recorded. The results revealed that the mitochondrial area, diameter, and perimeter were significantly lower and that the number of small mitochondria was greater in the livers of the KET group than in those of the CON group (Fig. 2A and B). The Dynamin-Related Protein 1/Mitochondrial Fission Factor (DRP1/MFF) signaling pathway plays an important role in mitochondrial fission. Therefore, we assessed the expression of key mitochondrial fission-related molecules, including DRP1, mitochondrial fission 1 protein (FIS1), MFF, mitochondrial dynamics protein 49 (MID49), and dynamin-2 (DYN2), via immunofluorescence staining and qRT-PCR. The results indicated that the expression of hepatic DRP1/MFF signaling pathway-related molecules was significantly greater in the KET group than in the CON group (Fig. 2C, D, and Fig. S1A). These results suggested that the activation of the DRP1/MFF pathway leads to aberrant mitochondrial fission, inducing excessive mitochondrial fragmentation and contributing to mitochondrial dysfunction.

The intracellular ROS levels were detected via the fluorescence probe DHE. The ROS content was significantly greater in the KET group than in the CON group (Fig. 2E and Fig. S1B). Moreover, the expression and concentration of hepatic 8-OHdG, a hallmark of oxidative stress-induced DNA damage, were significantly increased in the KET group, as determined by immunofluorescence and ELISA (Fig. 2F, G, and Fig. S1C). We subsequently performed immunofluorescence double staining to assess the expression of DNA oxidative damage biomarkers (γH2AX) and apoptosis (TUNEL) in the liver. Compared to the CON group, the KET group presented a significant increase in TUNEL-positive nuclei and γH2AX-positive nuclei (Fig. 2H). Moreover, the percentage of double-positive nuclei for TUNEL and γH2AX was also significantly elevated (Fig. 2I), indicating severe oxidative damage in the livers of ketotic cows. These findings suggested that activation of the DRP1/MFF pathway leads to excessive mitochondrial fission, causing mitochondrial dysfunction and ROS production, which in turn induces hepatic oxidative damage and apoptosis in dairy cows with ketosis.

The nuclear factor erythroid 2-related factor 2/heme oxygenase 1 (Nrf2/HO-1) pathway is a key regulator of the antioxidative response. Immunofluorescence double staining revealed a significant reduction in the expression of hepatic Nrf2 and HO-1 in the KET group compared to their expression in the CON group (Fig. 2J). Additionally, the qRT-PCR results showed a downward trend in the mRNA expression of *Nrf2* and *HO-1* in the livers of the ketotic cows (Fig. 2K). The expression of Cu/Zn superoxide dismutase (SOD1), an important antioxidant enzyme for ROS scavenging, was also significantly lower in the livers of ketotic cows, as indicated by the results of immunofluorescence assays (Fig. 2L and M). The levels of antioxidative indicators (T-SOD, GSH-PX, CAT, and T-AOC) and oxidative stress products (MDA) were also determined. The KET group presented significantly higher MDA levels and markedly lower levels of antioxidative indicators (T-SOD, GSH-PX, CAT, and T-AOC) in the liver (Fig. 2N), indicating a weakened antioxidant capacity and intracellular ROS scavenging in dairy cows with ketosis.

### Proteomic analysis reveals the activation of extracellular matrix (ECM)-related functions and the NF-κB pathway in the livers of ketotic cows

**Fig. 3:**
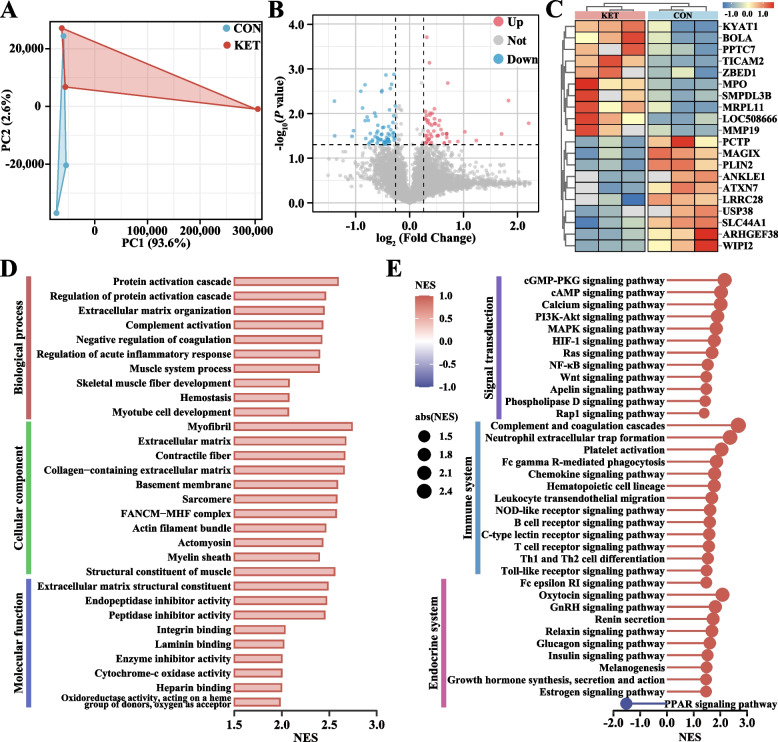
Analysis of liver proteomics data. **A** Principal component analysis (PCA) plot (*n* = 3). **B** Volcano plot of differentially expressed proteins (DEPs). **C** Heatmap of top 10 upregulated and downregulated DEPs. **D** GO enrichment analysis was conducted via gene set enrichment analysis (GSEA). **E** KEGG enrichment pathway analysis was conducted via GSEA. NES represents the normalization of enrichment scores

A proteomic analysis was performed to identify the key proteins involved in hepatic macrophage activation in ketotic cows. Principal component analysis (PCA) revealed complete separation between the CON and KET groups (Fig. 3A). A volcano plot was made to show the DEP expression patterns, including 73 upregulated DEPs and 65 downregulated DEPs (Fig. 3B). The top 10 upregulated and downregulated DEPs are presented in Fig. 3C and Table S6 (ranked in |Log_2_ FC|). These DEPs were involved in mitochondrial metabolism (mitochondrial ribosomal protein L11, MRPL11), inflammation (myeloperoxidase, MPO), and fibrosis (matrix metallopeptidase 19, MMP19). Next, gene set enrichment analysis (GSEA) was performed for the proteins in the CON and KET groups using the GO and KEGG databases. The obtained GO and KEGG gene sets were scored with enrichment scores (ES), after which the ES were normalized (NES) and subjected to a permutation test. Finally, according to the ES and *P* values, we identified the significantly enriched GO functions and KEGG pathways in the KET group. The results of the GO analysis revealed that the DEPs were enriched mainly in ECM organization, ECM, collagen-containing ECM, and ECM structural constituents (Fig. 3D and Table S7). The KEGG enrichment analysis revealed 133 differential pathways at level 3 (*P* < 0.05). These pathways are distributed mainly in the immune system (10.5%), signal transduction (9.0%), and endocrine system (7.5%) (Table S8). The PI3 K-Akt signaling pathway, HIF-1 signaling pathway, NF-κB signaling pathway, neutrophil extracellular trap formation pathway, and Toll-like receptor signaling pathway are involved in liver inflammation and fibrosis (Fig. 3E and Table S9).

**Fig. 4:**
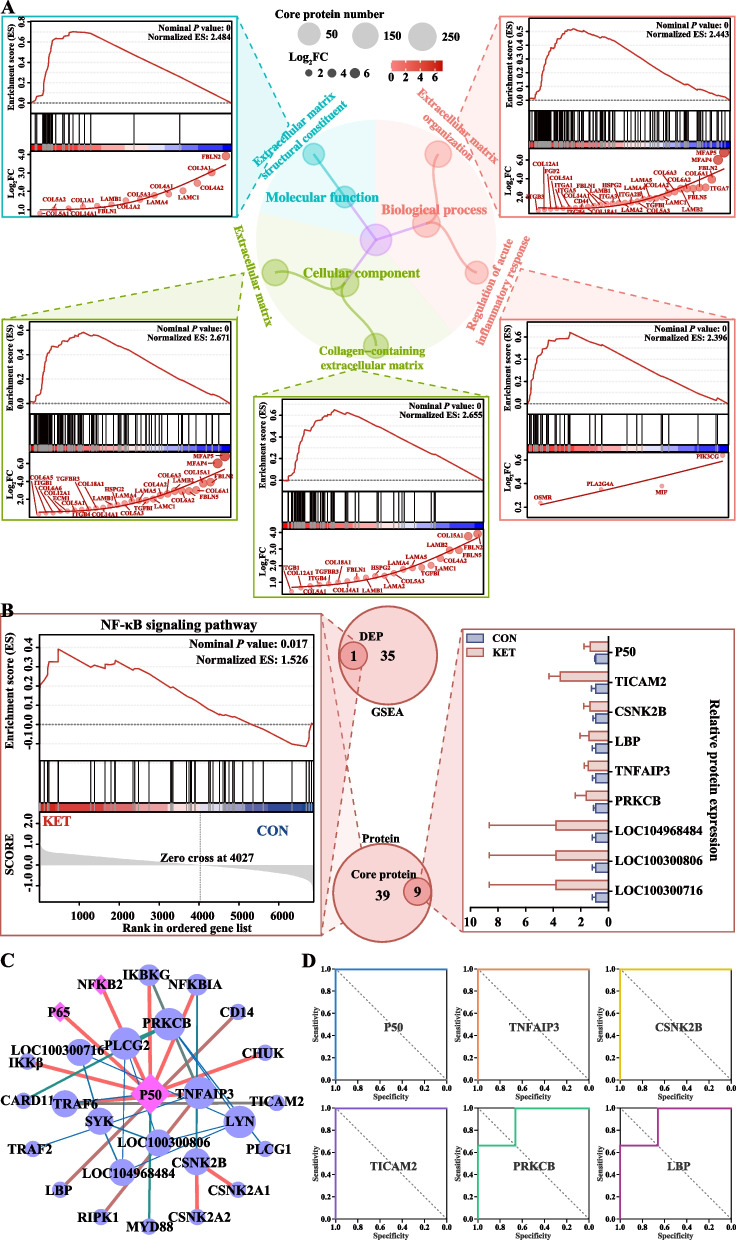
Identification of extracellular matrix (ECM)-related functions and the NF-κB signaling pathway. **A** GSEA of ECM-related functions. The core ECM-related pathways are shown in the center. The size of the circle represents the number of core proteins, and the size and color of each circle indicate fold changes in protein expression. **B** GSEA results for the NF-κB signaling pathway and the expression patterns of core proteins. The overlapping pathway between the GSEA and differential enrichment analysis results was the NF-κB signaling pathway; DEP, differentially enriched pathway. The relative expression of core proteins in the NF-κB signaling pathway is shown on the right. **C** PPI network of the NF-κB pathway from the STRING database. Lines represent protein-protein interactions; circle size reflects protein abundance; purple diamonds indicate transcription factors; blue circles represent proteins. **D** Receiver operating characteristic (ROC) analysis of the six annotated core proteins in the NF-κB signaling pathway. The area under the curve (AUC) indicates the potential of a protein as a biomarker

According to the enriched GO pathways and published documents, ECM organization, the ECM, the collagen-containing ECM, the ECM structural constituent, and the regulation of the acute inflammatory response may be closely related to liver fibrosis in ketotic cows (Fig. 4A). The corresponding GSEA plot revealed that the core proteins were upregulated in the KET group. Moreover, several profibrogenic proteins were upregulated in the KET group (Fig. 4A). For example, members of the collagen family, including COL1, 2, 3, 4, 5, 6, 12, 14, 15, and 18, were identified. Among the 36 key pathways identified by GSEA, the NF-κB signaling pathway was the only pathway with significant enrichment of DEPs.

Further analysis of the NF-κB signaling pathway revealed that among the 39 protein sets, nine core proteins play crucial roles. Protein expression profiling revealed that the relative expression of nine core proteins (P50, TICAM2, CSNK2B, LBP, TNFAIP3, PRKCB, LOC104968484, LOC100300806, and LOC100300716) in the NF-κB pathway was higher in the KET group than in the CON group (Fig. 4B). The interaction of core proteins of the NF-κB signaling pathway is shown in a protein-protein interaction (PPI) network, and P50 showed the strongest interaction with other proteins (Fig. 4C). The receiver operating characteristic (ROC) curves showed that the six annotated core proteins of the NF-κB signaling pathway may be highly accurate biomarkers of liver fibrosis in the KET group (Fig. 4D). These results indicated that the enrichment of ECM-related functions and the activation of the NF-κB pathway in the livers of ketotic cows are closely associated with fibrogenesis.

### Hepatic cytokine network dysregulation in dairy cows with ketosis

**Fig. 5:**
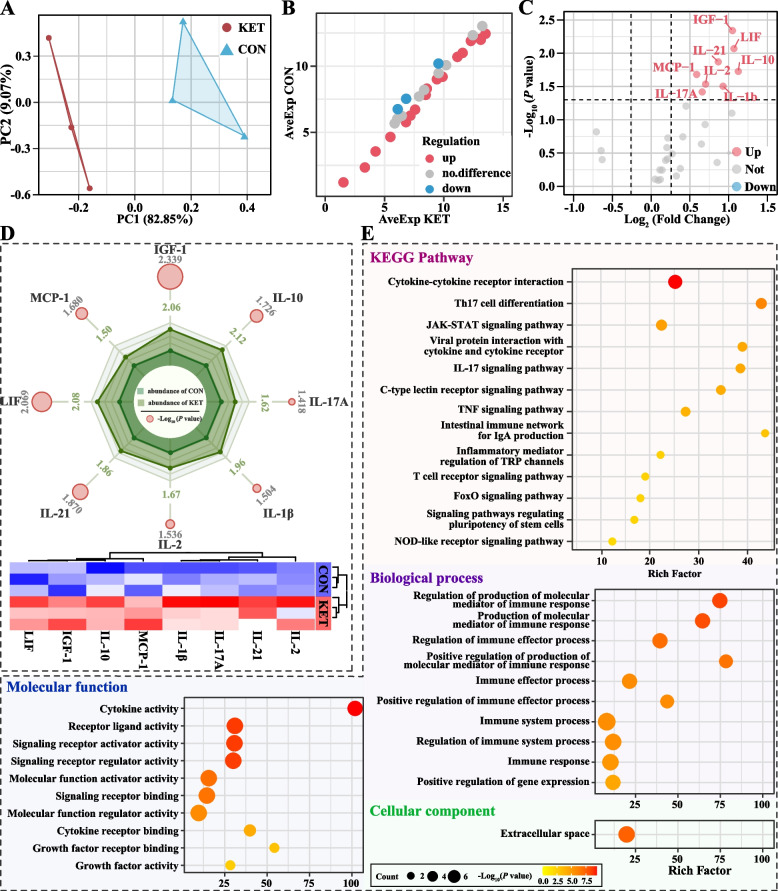
Hepatic cytokine network dysregulation in dairy cows with ketosis. **A** PCA plot of the hepatic cytokine expression profile (*n* = 3). **B** Scatter plot showing the cytokine expression patterns. AveExp represents the average expression of cytokines. **C** Volcano plot of cytokine alterations in liver tissue as measured by the Multiplex Quantibody Bovine Cytokine Array Q30. The log2 (fold change) represents the difference in cytokine expression between the two groups. The −log_10_ (*P*-value) indicates the significance of DECs. **D** Radar map and heatmap of DECs. The green numbers indicate the fold changes in the expression of cytokines, while the circle size represents the −log_10_ (*P* value). Shading indicates the average abundance in the CON and KET groups in the radar map. The heatmap displays the expression patterns of DECs. **E** KEGG and GO enrichment pathways of DECs. GO functions are categorized into biological process (BP), molecular function (MF), and cellular component (CC) categories. The circle size represents the number of cytokines enriched in the pathway, while the circle color indicates −log_10_ (*P* value)

Given the crucial role of macrophages in liver cytokine expression, Multiplex Quantibody Bovine Cytokine Array Q30 was used to detect cytokine profiles in the livers of ketotic cows. PCA results revealed a clear separation of the cytokine profiles of the KET and CON dairy cows (Fig. 5A). A scatter plot was constructed to visualize the cytokine expression patterns (Fig. 5B). The DECs were displayed as volcano plots and heatmaps. Compared to those in the CON group, the expression of eight cytokines differed: interleukin-2 (IL-2), interleukin-21 (IL-21), interleukin-17 A (IL-17A), interleukin-1β (IL-1β), monocyte chemoattractant protein-1 (MCP-1), interleukin-10 (IL-10), insulin-like growth factor 1 (IGF-1), and leukemia inhibitory factor (LIF) (Fig. 5C and D, Table S10). Normalized data for the levels of these cytokines in the KET and CON groups are expressed as a radar plot (Fig. 5D). The radar map showed that certain cytokines were related to macrophage recruitment and liver inflammation, particularly IL-2, IL-21, IL-17A, IL-1β, MCP-1, and LIF.

To better understand the function of DECs, GO and KEGG enrichment analyses were conducted. GO annotations were conducted to analyze the functional information on the molecular functions (MFs), biological processes (BP), and cellular components (CC) related to DECs (Fig. 5E and Table S11). In the MF category, “cytokine activity”, “receptor ligand activity”, and “signaling receptor activator activity” were the main enriched terms. In the BP category, “regulation of production of molecular mediator of immune response”, “production of molecular mediator of immune response” and “regulation of immune effector process” were significantly enriched. Particularly, we found that DECs were mainly enriched in the “extracellular space” in the CC, which may contribute to the progression of liver fibrosis. The results of the KEGG annotation revealed that certain KEGG pathways related to inflammation-related pathways, including “T helper 17 (Th17) cell differentiation”, “JAK-STAT signaling pathway”, “IL-17 signaling pathway”, and “TNF signaling pathway”, were the main enriched pathways (Fig. 5E and Table S12). These results suggested that the dysregulation of cytokine networks contributes to liver inflammation, creating a proinflammatory microenvironment in the livers of ketotic cows.

### NF-κB activation induced a proinflammatory shift in hepatic macrophages in dairy cows with ketosis

**Fig. 6:**
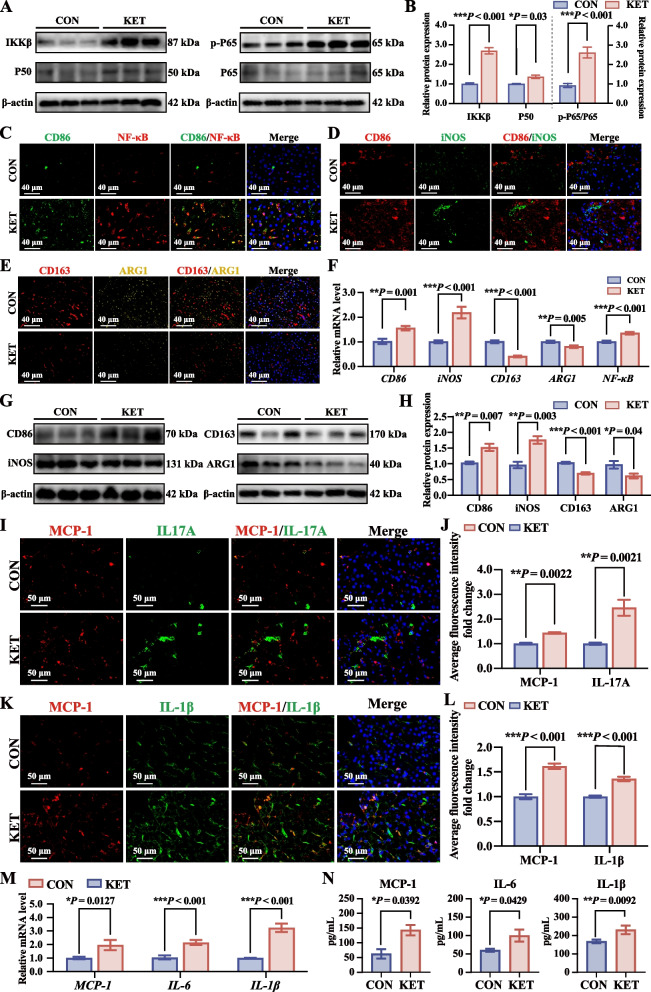
NF-κB activation triggered a proinflammatory shift in hepatic macrophages from dairy cows with ketosis. **A** Western blotting analysis of the expression of NF-κB pathway genes (IKKβ, P50, p-P65, and P65). **B** Relative protein abundance of IKKβ, P50, p-P65, and P65 (*n* = 5). **C** Immunofluorescence double-stained images of CD86 (green) and nuclear factor kappa-B P65 (NF-κB, red) are shown. **D** Immunofluorescence double-stained images of the M1 macrophage markers CD86 (red) and inducible nitric oxide synthase (iNOS, green). **E** Immunofluorescence double-stained images of the M2 macrophage markers CD163 (red) and arginase 1 (ARG1, yellow). **F** qRT-PCR analysis was conducted to determine the mRNA expression levels of *CD86*, *iNOS*, *CD163*, *ARG1*, and *NF-κB* (*n* = 5). **G** Western blotting analysis of the protein expression of M1 and M2 markers. **H** Relative protein abundance of M1 (CD86 and iNOS) and M2 (CD163 and ARG1) markers (*n* = 5). **I** Immunofluorescence images of monocyte chemoattractant protein-1 (MCP-1, red) and interleukin-17 A (IL-17A, green) double-staining. **J** Average fold change in the fluorescence intensity of MCP-1 and IL-17A (*n* = 5). **K** Immunofluorescence images of MCP-1 (red) and interleukin-1β (IL-1β, green) double-staining. **L** Average fold change in the fluorescence intensity of MCP-1 and IL-1β (*n* = 5). **M** qRT-PCR analysis was conducted to determine the mRNA expression levels of *MCP-1*, *IL-6*, and *IL-1β* (*n* = 5). **N** Concentrations of MCP-1, IL-6, and IL-1β in the liver (*n* = 5). The values are expressed as the mean ± SEM. Statistical analysis was performed by conducting unpaired Student’s *t*-tests (two-tailed); ^*^P < 0.05, ^**^P < 0.01, and ^***^P < 0.001

Activation of the NF-κB signaling pathway is closely associated with the activation of macrophages. We further analyzed the abundance of core proteins of the NF-κB signaling pathway (IKKβ, P50, p-P65, and P65) via Western blotting. The hepatic abundance of IKKβ, P50, and p-P65/P65 proteins was greater in the KET group than in the CON group (Fig. 6A and B). Immunofluorescence double staining revealed higher expression of proinflammatory M1 macrophage-associated markers (CD86 and iNOS) and lower expression of M2 anti-inflammatory macrophage markers (CD163 and ARG1) in the KET group than in the CON group (Fig. 6C, D, and Fig. S1D and E). Similarly, the mRNA expression levels of *CD86* and *iNOS* were significantly higher, whereas the *CD163* and *ARG1* expression levels were significantly lower in the KET group (Fig. 6F). Additionally, costaining for NF-κB and the M1 macrophage marker CD86 was significantly higher in the KET group (Fig. 6E), suggesting that the activation of NF-κB drives the M1 polarization of liver macrophages in dairy cows with ketosis. Consistent with the immunofluorescence results, the mRNA expression levels of hepatic *NF-κB* were significantly higher in the KET group (Fig. 6F). Further analysis of M1 and M2 macrophage markers by Western blotting revealed that the protein abundance of CD86 and iNOS (M1 marker) was greater in KET cows than in CON cows, but the protein abundance of CD163 and ARG1 (M2 marker) was lower (Fig. 6G and H). These findings indicated that NF-κB activation induces a proinflammatory shift in the macrophage phenotype, disrupting the balance between M1 and M2 macrophages in ketotic cows.

Activated M1 macrophages release various inflammatory cytokines, which dysregulate the proinflammatory microenvironment in the liver. Immunofluorescence double staining revealed that the expression of MCP-1, IL-17A, MCP-1, and IL-6 was considerably higher in the livers of ketotic cows (Fig. 6I). Additionally, compared to those in the CON group, the contents and mRNA expression levels of MCP-1, IL-6, and IL-1β measured by qRT-PCR and ELISA were significantly greater in the KET group (Fig. 6M and N). These results confirmed that the activation of NF-κB induces a proinflammatory shift in macrophages, which contributes to cytokine network dysregulation in the livers of ketotic cows.

### Hepatic stellate cell (HSC) activation leads to ECM deposition and fibrosis

**Fig. 7:**
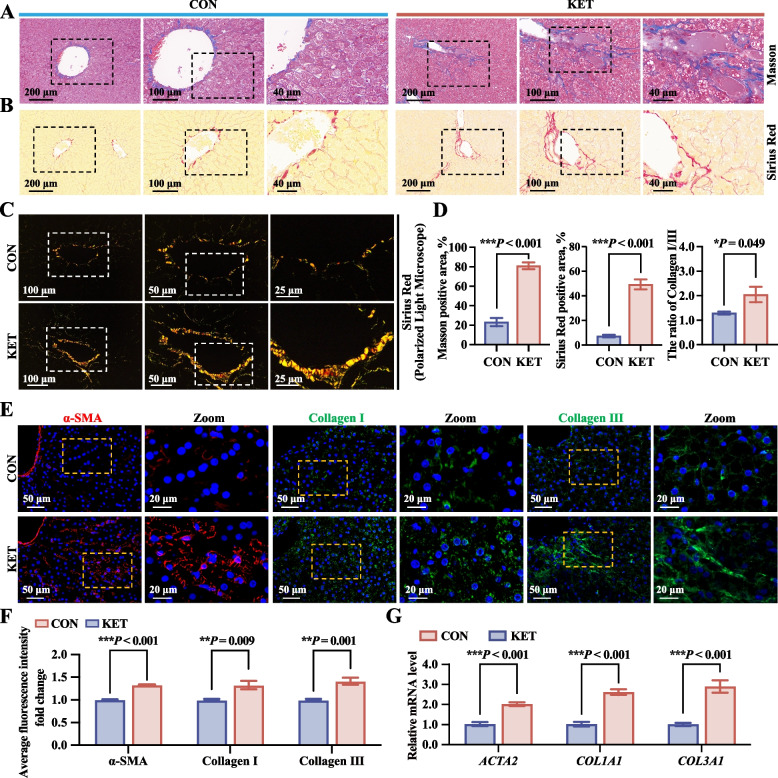
HSC activation led to ECM deposition and fibrosis. **A** Masson staining of liver tissue. **B** Sirius red staining of liver tissue under a light microscope. **C** Sirius red staining of liver tissue under a polarized light microscope. **D** Quantitative analysis of the percentage of Masson-positive area, Sirius red-positive area, and the ratio of collagen I/collagen III (*n* = 5). **E** Immunofluorescence analysis of the expression of HSC activation markers (α-smooth muscle actin, α-SMA) and ECM deposition markers (collagen I and collagen III). **F** Average fold changes in the fluorescence intensities of α-SMA, collagen I, and collagen III (*n* = 5). **G** qRT-PCR analysis was conducted to determine the mRNA expression levels of *ACTA2*, *COL1A1*, and *COL3A1* (*n* = 5). The values are expressed as the mean ± SEM. Statistical analysis was performed by conducting unpaired Student’s *t*-tests (two-tailed); ^*^P < 0.05, ^**^P < 0.01, and ^***^P < 0.001

A dysregulated proinflammatory microenvironment is closely associated with the progression of liver fibrosis through the activation of HSCs. Masson staining and Sirius red staining were conducted to visually quantify collagen fibers. Substantial accumulation of collagen fiber (blue collagen deposition in the hepatic sinusoids and central vein) was detected via Masson’s trichrome staining in the KET group compared to that in the CON group (Fig. 7A and D). Sirius red staining revealed a significant increase in collagen deposition in the livers of dairy cows under an ordinary light microscope (Fig. 7B and D). Under polarized light, primary collagen I (red and yellow staining) and a few collagen III (green staining) depositions in the liver were higher in the KET group than in the CET group (Fig. 7C and D). Moreover, immunofluorescence analysis showed that the expression of the HSC activation marker (α-SMA) and ECM deposition markers (collagen I and III) in liver tissues was significantly higher in the KET group than in the CON group (Fig. 7E and F); additionally, the mRNA levels of the ECM genes collagen type I alpha 1 chain (*COL1A1*), collagen type III alpha 1 chain (*COL3A1*), and the HSC activation marker actin alpha 2 (*ACTA2*, the gene encoding α-SMA) were also higher in the KET group, as determined by qRT-PCR analysis (Fig. 7G). These findings indicated that proinflammatory macrophages contribute to the activation of HSCs, leading to ECM deposition and contributing to liver fibrosis in dairy cows with ketosis.

### Correlation analysis between macrophage proinflammatory activation markers and liver damage features

**Fig. 8:**
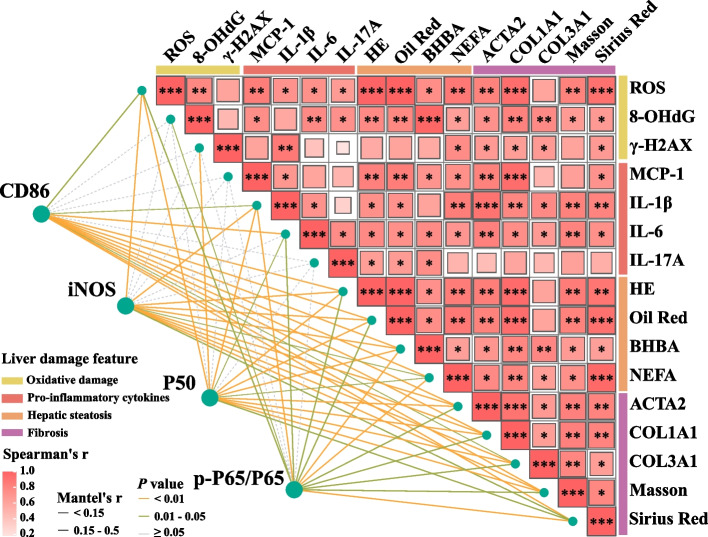
Relationships between macrophage proinflammatory activation markers and liver damage features. Pairwise comparisons of liver damage feature indicators are shown with a color gradient representing Spearman’s coefficient. Asterisks (*) indicate significance (^*^P < 0.05, ^**^P < 0.01, and ^***^P < 0.001). Macrophage proinflammatory activation markers were associated with each indicator, with the width of the lines reflecting the strength of the correlation, as determined by the Mantel test. The color of the lines represents significance: orange lines indicate *P* < 0.01, and green lines indicate 0.01 < *P* < 0.05

Correlation analysis revealed that the expression levels of macrophage proinflammatory activation markers are related to liver damage features, including oxidative damage, proinflammatory cytokines, hepatic steatosis, and fibrosis indicators. The levels of macrophage proinflammatory activation markers (CD86, iNOS, P50, and p-P50/P50) were significantly correlated with the levels of oxidative damage markers (ROS), proinflammatory cytokines (IL-1β), hepatic steatosis indicators (HE, Oil Red, BHBA, and NEFA), and fibrosis markers (*ACTA2*, *COL1A1*, and *COL3A1*, Masson and Sirius Red) (Fig. 8). These findings highlighted that the proinflammatory activation of macrophages may contribute to liver damage in dairy cows with ketosis.

## Discussion

The causes and development of liver fibrosis are complex, and our findings revealed that the proinflammatory shift in macrophages during ketosis may be a key factor in inducing liver fibrosis. In this study, an integrated proteomics and cytokine array approach was used to reveal the changes in the expression of proteins and cytokines associated with liver fibrosis in ketotic cows. The content and expression of markers associated with collagen and fibers, which are the main components of the ECM and a distinctive feature of fibrosis, increased in the livers of ketotic cows. Additionally, a cytokine array revealed that the cytokine networks were dysregulated, which further contributed to liver inflammation and fibrosis. These findings indicated that the interactions of fibrogenic proteins and cytokines are crucial factors driving liver fibrosis.

Ketosis is a metabolic disorder in dairy cattle and is closely associated with inflammatory and fibrotic processes in the liver [1, 2]. These pathological changes can decrease the efficiency of dairy production and threaten the health and welfare of cows. High blood concentrations of NEFAs and BHBA play crucial roles in the pathogenesis of hepatic injury in dairy cows with ketosis [1, 8]. High levels of NEFAs and BHBA exhibit strong lipotoxicity, inducing hepatocellular oxidative stress and impairing mitochondrial function, further leading to the deposition of lipid droplets and inflammation [7, 10–12]. In this study, we preliminarily revealed histopathological changes, including hepatic steatosis, infiltration of inflammatory cells, an increase in lipid deposition, and a decrease in glycogen expression, in dairy cows with ketosis. This finding is further supported by the significantly increased serum NEFA, BHBA, and liver function-associated enzyme activity (AST, ALT, and GGT) levels, which revealed that liver function was significantly impaired. We also detected abnormal mitochondrial fission, oxidative imbalance, inflammation, apoptosis, and fibrotic lesions in ketotic cows. Proteomic analysis revealed that multiple ECM functions were enriched and that the NF-κB signaling pathway was significantly different in the livers of ketotic cows. A cytokine array revealed dysregulated cytokine networks, followed by macrophage proinflammatory polarization and overactivation of the NF-κB signaling pathway. We identified the multifactorial and multicellular interaction effects driving liver fibrosis in dairy cows with ketosis by conducting multiomics mechanistic analysis and identified the molecular mechanism involved in macrophage polarization.

Mitochondrial dysfunction is a key factor in the occurrence and progression of hepatic injury in ketotic cows and is often accompanied by excessive ROS production and the release of inflammatory cytokines [7, 10, 29]. Mitochondrial fission, mediated by the DRP1/MFF signaling pathway, plays a pivotal role in maintaining mitochondrial function. Disruption of this pathway can lead to mitochondrial dysfunction and ROS overproduction [16, 17, 30]. DRP1, a key regulator of mitochondrial fission, is recruited to the outer mitochondrial membrane by adaptor proteins, including FIS1, MID49, and MFF; this facilitates the formation of DRP1 oligomers and subsequent membrane constriction [14]. DYN2 then interacts with DRP1 to complete membrane scission [31]. Fission segregates damaged mitochondria, which contain high levels of ROS and low membrane potential [32]. The overexpression and activation of DRP1 can induce aberrant mitochondrial fission and excessive fragmentation, significantly promoting mitochondrial dysfunction and ROS generation [33, 34]. Moreover, DRP1-mediated abnormal mitochondrial fission facilitates the deposition of lipid droplets in hepatic cells, which contributes to the progression of nonalcoholic fatty liver disease-like changes [35]. Our results revealed that DRP1 was overexpressed in the livers of ketotic cows and that the DRP1/MFF signaling pathway was significantly activated. The activation of the mitochondrial DRP1/MFF pathway, along with the increased number of small mitochondria, indicated excessive mitochondrial fission in dairy cows with ketosis. Excessive mitochondrial fission further exacerbated mitochondrial dysfunction, contributed to intracellular ROS overproduction, and was involved in hepatic oxidative damage in dairy cows with ketosis.

Oxidative stress occurs when there is an imbalance between ROS production and antioxidant regulation [36]. The Nrf2/HO-1 pathway is a classic antioxidant signaling mechanism that controls the expression of key components of the antioxidant system [37]. In response to high levels of ROS, Nrf2 translocates into the nucleus and binds to antioxidant response elements, thereby increasing the transcription of antioxidant enzymes (including SOD, CAT, GSH-PX, and HO-1) to restore redox balance [38]. Excessive BHBA can increase ROS production and disrupt the Nrf2/HO-1 pathway in bovine macrophages in vitro [39]. In our study, the Nrf2/HO-1 signaling pathway was inhibited in the livers of ketotic cows in vivo, along with a decrease in the activity of downstream antioxidant enzymes; these changes decreased intracellular ROS scavenging capacity. Additionally, excess ROS production caused by aberrant mitochondrial fission inhibits the Nrf2/HO-1 signaling pathway, further exacerbating the progression of oxidative stress in the livers of ketotic cows.

Oxidative stress mediated by ROS is associated with macrophage activation [40]. Several studies have highlighted the critical role of ROS in inducing and maintaining M1 macrophage polarization [41–43]. Excessive ROS production probably triggers the phenotypic switch of hepatic macrophages from an anti-inflammatory (M2) state to a proinflammatory (M1) state, leading to hepatic inflammation and insulin resistance [43, 44]. The M1 polarization of macrophages drives hepatic steatosis and inflammation [45]. M1 macrophages aggravate the severity of nonalcoholic fatty liver disease by promoting inflammatory responses and cytokine secretion, which contribute to hepatic steatosis and inflammation [46], whereas M2-polarized macrophages can attenuate liver injury through their anti-inflammatory effects and ability to promote tissue repair [47]. We found a significant increase in the expression of hepatic M1 macrophage markers and a decrease in the expression of M2 macrophage markers in dairy cows with ketosis. This imbalance between M1 and M2 macrophages may play a role in the development of hepatic steatosis and inflammation. Therefore, we speculated that intracellular ROS accumulation triggers the transformation of macrophages to proinflammatory M1 macrophages, further driving hepatic steatosis and inflammation in ketotic cows.

Multi-omics could provide an integrated view of biological information for comprehensively understand the elaborate mechanism of diseases for better investigating targeted treatment. In this study, we performed proteomics and cytokine array analyses to elucidate the underlying molecular mechanisms of liver fibrosis in ketotic cows. Our proteomics results revealed that multiple ECM functions were enriched and that the NF-κB signaling pathway was significantly different in the livers of ketotic cows. Moreover, a cytokine array revealed dysregulated cytokine networks accompanied by macrophage proinflammatory polarization and overactivation of the NF-κB signaling pathway. Therefore, we speculated that the activation of NF-κB promotes the release of proinflammatory mediators, further exacerbating the deposition of ECM in the livers of ketotic cows. NF-κB is a key transcription factor involved in regulating immunity and inflammatory responses [48]. The activation of NF-κB signaling relies on three important components: NF-κB, the inhibitor of NF-κB (IκB), and the IκB kinase complex (IKK). Upstream signals activate the IKK complex, largely through IKKβ, and phosphorylate IκB, resulting in its degradation by the proteasome. Subsequently, canonical NF-κB family members, including NF-κB p50 and NF-κB p65, translocate to the nucleus to initiate the activation of target genes [49, 50]. In our study, proteomics analysis revealed that the NF-κB signaling pathway was the only significantly different pathway among the 36 key pathways identified by the GSEA. The NF-κB signaling pathway is a potential modulator of macrophage M1 polarization and proinflammatory cytokine production [51]. To investigate the regulatory mechanism of hepatic macrophage polarization in dairy cows with ketosis, we assessed the related molecules in the NF-κB signaling pathway. Consistent with the proteomics results, the significantly increased expression of IKKβ and NF-κB p50, along with the increased phosphorylation levels of NF-κB p65, indicate that the hepatic NF-κB signaling pathway is overactivated during ketosis. Moreover, the upregulation of M1 macrophage polarization markers (CD86 and iNOS) and the downregulation of M2 macrophage polarization markers (CD163 and ARG1) indicated that the activated NF-κB signaling pathway drives the phenotypic shift in hepatic macrophages toward a proinflammatory state. ROS can regulate NF-κB activity by directly influencing NF-κB heterodimers (p50 and p62) or the upstream of IKK complex [52]. Several studies have also revealed the role of ROS in NF-κB activation, further promoting the expression of proinflammatory genes in macrophages [41–43]. In this study, the increased polarization of M1 macrophages was accompanied by the overactivation of NF-κB, suggesting that high levels of ROS activate the NF-κB signaling pathway in macrophages, inducing a shift toward classically activated (M1) macrophages.

Macrophages are key regulators of liver inflammation that control the progression of liver fibrosis by producing proinflammatory cytokines and activating HSCs [53–55]. Cytokine network dysregulation is an adverse effect of macrophage activation, where it often leads to liver inflammation [56]. We characterized the cytokine profile in the livers of cows with ketosis. Cytokine profile analysis revealed that most proinflammatory cytokines, such as MCP-1, IL-1β, IL-6, LIF, and IL-17A, were considerably upregulated in the livers of ketotic cows and involved in inflammation-related signaling pathways. LIF is a novel inflammatory modulator that plays a crucial role in driving the recruitment of macrophages to inflamed sites and aggravating the inflammatory response [57]. M1 macrophages mainly produce MCP-1, IL-1β, and IL-6, which promote hepatic inflammation, macrophage recruitment, and HSC activation; they also mediate the upregulation of fibrogenic genes [53, 58, 59]. The differentiation of Th17 cells and the IL-17 signaling pathway were significantly enriched in this study. IL-17A, expressed by the Th17 cell lineage, has a strong profibrogenic effect by directly activating HSCs to express collagen type I and promoting their activation into fibrogenic myofibroblasts [59, 60]. M1 macrophage polarization induces a proinflammatory microenvironment and promotes the differentiation of Th17 cells and activation of HSCs, further leading to the accumulation of collagen fibers in the ECM [61, 62]. Along with these proinflammatory cytokines, we found that the expression of IL-10, an anti-inflammatory cytokine that can limit immune cell activation [63], increased significantly, as determined by a cytokine array. Increased expression of IL-10 in ketotic cows may be a compensatory response to counteract hepatic inflammation. In this study, we demonstrated the occurrence of pathogenic fibrosis in the livers of cows with ketosis, as indicated by Sirius Red and Masson staining, which was confirmed by a substantial increase in the expression of ECM components (collagen I and III) and an HSC activation marker (α-SMA). These findings indicated that M1 polarization of macrophages induces a hepatic proinflammatory microenvironment and activation of HSCs, further leading to the accumulation of ECM and ultimately contributing to the progression of liver fibrosis.

## Conclusion

**Fig. 9:**
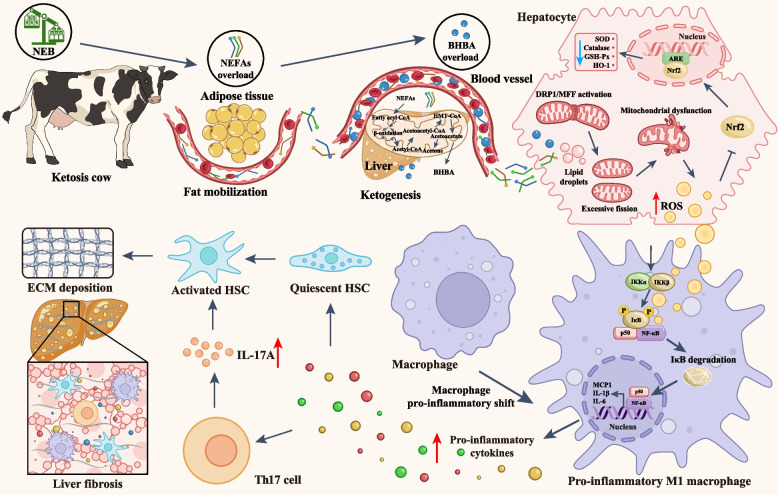
Schematic diagram of the macrophage proinflammatory shift that drive liver fibrosis in dairy cows with ketosis. Excessive mitochondrial fission, along with the inhibition of the Nrf2/HO-1 pathway, increases hepatic reactive oxygen species (ROS) production. The accumulation of ROS subsequently triggers NF-κB activation, promoting a proinflammatory shift in macrophages, which in turn leads to hepatic stellate cell (HSC) activation. This cascade results in the deposition of the extracellular matrix (ECM) and the progression of liver fibrosis in dairy cows with ketosis. DRP1, Dynamin-Related Protein 1; MFF, Mitochondrial Fission Factor; Nrf2, Nuclear Factor Erythroid 2-Related Factor 2; HO-1, Heme Oxygenase 1

To summarize, we identified the multifactorial and multicellular interaction effects driving liver fibrosis in dairy cows with ketosis using integrated proteomics and cytokine array approaches. We also analyzed the molecular mechanism involved in the macrophage proinflammatory shift. We found that excessive mitochondrial fission induced by the activation of the DRP1/MFF pathway contributes to hepatic ROS production, subsequently inhibits the Nrf2/HO-1 pathway, and weakens intracellular ROS scavenging. The accumulation of ROS triggers the activation of NF-κB, inducing a proinflammatory shift in macrophages, further resulting in cytokine network dysregulation and liver inflammation. The proinflammatory microenvironment caused by M1 macrophages induced HSC activation, leading to the deposition of ECM in the liver and fibrosis in dairy cows with ketosis (Fig. 9). Our findings provided new insights into the development of liver fibrosis in dairy cows with ketosis.

## Supplementary Information

Supplementary Material 1: Table S1 The basal diet formulation. Table S2 The basic characteristics of the normal and ketotic cows. Table S3 The primer sequences. Table S4 Antibodies used in immunofluorescence staining. Table S5 Antibodies used in western blotting. Table S6 The top 10 upregulated and downregulated DEPs. Table S7 The significantly enriched TOP 10 GO terms by GSEA. Table S8 The distributed of level 3 pathway by KEGG enrichment analysis. Table S9 The significantly enriched KEGG pathways by GSEA. Table S10 The cytokine alteration in liver tissue between KET group and CON group. Table S11 GO enrichment terms of DECs. Table S12 KEGG enrichment pathway of DECs. Fig. S1 Average fluorescence intensity analysis of immunofluorescence sections in the liver in dairy cows with ketosis.

## Data Availability

The datasets produced or analyzed during the current study are available from the corresponding author on reasonable request.

## Abbreviations

ALT: Alanine aminotransferase
ARE: Antioxidant response elements
ARG1: Arginase 1
AST: Aspartate aminotransferase
BHBA: β-Hydroxybutyric acid
DRP1: Dynamin-related protein 1
DYN2: Dynamin-2
ECM: Extracellular matrix
FIS1: Fission protein 1
GSEA: Gene set enrichment analysis
GSH-Px: Glutathione peroxidase
HO-1: Heme oxygenase 1
HSCs: Hepatic stellate cells
IGF-1: Insulin-like growth factor 1
iNOS: Inducible nitric oxide synthase
LIF: Leukemia inhibitory factor
MCP-1: Monocyte chemoattractant protein 1
MDA: Malondialdehyde
MFF: Mitochondrial fission factor
MID49: Mitochondrial dynamics proteins of 49 kDa
NEB: Negative energy balance
NEFAs: Nonesterified fatty acids
NF-κB: Nuclear factor kappa-B
Nrf2: Nuclear factor erythroid 2-related factor 2
ROC: Receiver operating characteristic
ROS: Reactive oxygen species
SOD1: Cu/Zn superoxide dismutase
T-AOC: Total antioxidant capacity
Th17: T helper 17
T-SOD: Total superoxide dismutase
8-OHdG: 8-Hydroxydeoxyguanosine
