# Supplementary material for: Proteomics and cytokine array jointly reveal the role of macrophage proinflammatory shift in liver fibrosis in dairy cows with ketosis
Source: J Anim Sci Biotechnol. 2025 Jul 8;16:97. doi: 10.1186/s40104-025-01219-4 (PMC12236012; doi:10.1186/s40104-025-01219-4)
Supplement: Supplementary file 1 — Supplementary Material 1: Table S1 The basal diet formulation. Table S2 The basic characteristics of the normal and ketotic cows. Table S3 The primer sequences. Table S4 Antibodies used in immunofluorescence staining. Table S5 Antibodies used in western blotting. Table S6 The top 10 upregulated and downregulated DEPs. Table S7 The significantly enriched TOP 10 GO terms by GSEA. Table S8 The distributed of level 3 pathway by KEGG enrichment analysis. Table S9 The significantly enriched KEGG pathways by GSEA. Table S10 The cytokine alteration in liver tissue between KET group and CON group. Table S11 GO enrichment terms of DECs. Table S12 KEGG enrichment pathway of DECs. Fig. S1 Average fluorescence intensity analysis of immunofluorescence sections in the liver in dairy cows with ketosis. [file 40104_2025_1219_MOESM1_ESM.docx]

**Supplemental Table S1.** The basal diet formulation.

| Ingredient composition | Content | Chemical composition | Content |
| --- | --- | --- | --- |
| Leymus chinensis, kg | 1.47 | DM, % of TMR | 49.3 |
| Wheat straw, kg | 0.00 | Ash, % | 8.35 |
| New silage, kg | 66.01 | CP, % | 18.03 |
| Corn, kg | 9.05 | NDF, % | 32.19 |
| Corn germ meal, kg | 4.89 | NFC, % | 42.09 |
| Soybean meal, kg | 2.69 | Crude fat, % | 3.38 |
| Rapeseed meal, kg | 3.67 | Calcium, % | 0.90 |
| Cottonseed, kg | 3.67 | Phosphorus, % | 0.47 |
| DDGS, kg | 3.67 | Sodium, % | 0.49 |
| Premix^1^, kg | 4.88 | Potassium, % | 1.11 |
| Total, kg | 100 | Chlorine, % | 0.54 |
|  |  | Sulfur, % | 0.23 |
|  |  | Magnesium, % | 0.40 |
|  |  | DCAD^2^, meq/100g | 20.17 |
|  |  | ME, Mcal/kg | 2.65 |
|  |  | NE_L_, Mcal/kg | 1.71 |
|  |  | NEm, Mcal/kg | 1.71 |
|  |  | NEg, Mcal/kg | 1.12 |

DDGS = distillers dried grains with solubles; TMR = total mixed ration; DM = dry matter; CP = crude protein; NDF = neutral detergent fiber; NFC = non-fiber carbohydrates; DCAD = dietary anion cation difference; ME = metabolizable energy; NE_L_ = net energy for lactation; NEm = energy for maintainance; NEg = net energy for gain.

^1^Premix contained the following ingredients per kilogram of diet: vitamin A, 2500 IU; vitamin D3, 500 IU; vitamin E, 70 IU; Cu (as reagent grade blue copperas), 6.4 mg; Mn (as reagent grade manganese sulfate monohydrate), 15.20 mg; Zn (as reagent grade zinc sulfate heptahydrate), 51.80 mg; I (as feed grade calcium iodate), 0.60 mg; Se (as feed grade sodium selenite), 0.17 mg; Co (as reagent grade cobalt carbonate), 0.09 mg

^2^DCAD = (Na^+^ + K^+^) - (Cl^-^ + S^2-^)

**Supplemental Table S2.** The basic characteristics of the normal and ketotic cows^1^.

| Items^2^ | CON (*n* = 15) | KET (*n* = 15) | *P* value |
| --- | --- | --- | --- |
| BW (kg) | 622.5 ± 20.02 | 631.7 ± 15.85 | 0.7213 |
| Milk yield (kg/d) | 32.73 ± 1.697 | 28.39 ± 1.018 | 0.0368^*^ |
| DMI (kg/d) | 21.47 ± 1.612 | 18.93 ± 1.714 | 0.2908 |

^1^ Data are expressed as mean ± SEM.

^2^ BW = body weight; DMI = dry matter intake.

**Supplemental Table S3.** The primer sequences.

| Genes  (NCBI gene ID)^1^ | Genbank accession number | Sequences 5’-3’ (For = Forward, Rev = reverse) | Product size (bp) | Annealing temperature (℃) |
| --- | --- | --- | --- | --- |
| *GAPDH* (281181) | NM_001034034.2 | For CAAGTTCAACGGCACAGTCAAG  Rev ATACTCAGCACCAGCATCACCC | 121 | 60 |
| *DRP1* (540892) | NM_001046494.2 | For GCCTGTTACAAATGAAATGGTCC  Rev TAACCCACAAGCATCAGCAAAG | 94 | 60 |
| *FIS1* (615565) | NM_001034784.2 | For GCAGACAGAGCCACAGAACAAC  Rev CAGCAAGTCCGATGAGTCCAG | 146 | 60 |
| *DYN2* (511691) | NM_001099369.1 | For TCGGCGTCATCACCAAACTC  Rev GTTGACCACTCCAATGTAGCCTCT | 107 | 60 |
| *MFF* (506291) | NM_001428906.1 | For ACCGAAGGTATTAGTCAGCGAA  Rev GTCTTCATTATTTCCTGCTACCACA | 156 | 60 |
| *MID49* (515229) | NM_001077925.1 | For ACATTCGGGAGATGTAGCCC  Rev GGGAGAAGGCATCCAACACA | 100 | 60 |
| *Nrf2* (497024) | NM_001011678.2 | For CCATTCAGCCAGCACAACAC  Rev TGAAACGTAGCCGAAGAAACCT | 169 | 60 |
| *HO-1* (513221) | NM_001014912.1 | For CAAGAAGGTTTTAAGCTGGTGATG  Rev TGTAGACGGGGTTCTCCTTGTT | 94 | 60 |
| *SOD1* (281495) | NM_174615.2 | For GAGGCAAAGGGAGATACAGTCGT  Rev CTGTCACATTGCCCAGGTCTC | 199 | 60 |
| *CD86* (414345) | NM_001038017.2 | For GGCCAAGAGAAGCCCAATAATGT  Rev AAGTTAGCCAGCACTATCAGGTCAG | 197 | 60 |
| *iNOS* (282876) | NM_001076799.1 | For CTATGCCAGCCCTCAGATTACAAC  Rev TGAGGTGGATCTCCGTGGGT | 187 | 60 |
| *CD163* (533844) | NM_001163413.1 | For AGTGCTGTCAGTTTCTCTGGCTC  Rev CTGAACACTTGGTGACTCCATCTAC | 220 | 60 |
| *ARG1* (513608) | NM_001046154.1 | For AGGCTGGTCTGCTTGAGAAACT  Rev TCTTCTTGACTTCTGCCACCAC | 171 | 60 |
| *NF-κB* (508233) | NM_001080242.2 | For AGCACGGACACCACCAAGAC  Rev CTCCAGGTCCCGCTTCTTTAC | 231 | 60 |
| *MCP-1* (281043) | NM_174006.2 | For AGCCAGATGCAATTAACTCCCA  Rev TTGCTGCTGGTGACTCTTCTGT | 103 | 60 |
| *IL-6* (280826) | NM_173923.2 | For GAGAATTTCCTGCAGTTCAGCC  Rev GTTTCTGACCAGAGGAGGGA | 100 | 60 |
| *IL-1β* (281251) | NM_174093.1 | For GGCAACCGTACCTGAACCCA  Rev CACGATGACCGACACCACCT | 205 | 60 |
| *ACTA2* (515610) | NM_001034502.1 | For GTGATGGTGGGAATGGGACAG | 196 | 60 |
|  |  | Rev CCTCGGTGAGAAGGGTTGGAT |  |  |
| *COL1A1* (282187) | NM_001034039.2 | For TCCTTCTGGTCCTCGTGGTCTC | 156 | 60 |
|  |  | Rev TCGCCATCATCTCCGTTCTT |  |  |
| *COL3A1* (510833) | NM_001076831.1 | For CCAGTACGAAGCATATGATGTCAAG | 248 | 60 |
|  |  | Rev GTCTTCCTGATTCCCCATCTTTTC |  |  |
|  | | |  |  |

^1^NCBI = National Center for Biotechnology Information

**Supplementary Table S4.** Antibodies used in immunofluorescence staining.

| Primary  antibodies | Catalog | Vendor | Source | Dilution | Secondary antibodies |
| --- | --- | --- | --- | --- | --- |
| DRP1 | GB115659 | Servicebio | Rabbit | 1:300 | Cy3 conjugated Goat Anti-Rabbit IgG |
| FIS1 | 10269-1-ap | Proteintech | Rabbit | 1:200 | Cy3 conjugated Goat Anti-Rabbit IgG |
| MFF | GB114102 | Servicebio | Rabbit | 1:500 | Cy3 conjugated Goat Anti-Rabbit IgG |
| MID49 | GB115278 | Servicebio | Rabbit | 1:1000 | Cy3 conjugated Goat Anti-Rabbit IgG |
| DYN2 | GB114607 | Servicebio | Rabbit | 1:300 | Cy3 conjugated Goat Anti-Rabbit IgG |
| γH2AX | GB111841 | Servicebio | Rabbit | 1:200 | Cy3 conjugated Goat Anti-Rabbit IgG |
| SOD1 | 10956-1-ap | Proteintech | Rabbit | 1:200 | Alexa Fluor 488 conjugated Goat Anti-Rabbit IgG |
| 8-OHdG | sC-393871 | Santa | Mouse | 1:100 | Cy3 conjugated Goat Anti-mouse IgG |
| α-SMA | GB111364 | Servicebio | Rabbit | 1:200 | Cy3 conjugated Goat Anti-Rabbit IgG |
| Collagen Ⅰ | BA0325 | Boster | Rabbit | 1:300 | Alexa Fluor 488 conjugated Goat Anti-Rabbit IgG |
| Collagen Ⅲ | GB111629 | Servicebio | Rabbit | 1:300 | Alexa Fluor 488 conjugated Goat Anti-Rabbit IgG |

The antibodies information for fluorescent single labeling staining

| Primary antibodies | Catalog | Vendor | Source | Dilution | Secondary antibodies | Corresponding TSA |
| --- | --- | --- | --- | --- | --- | --- |
| Nrf2/HO-1 | GB113808/GB11549 | Servicebio/Servicebio | Rabbit/Rabbit | 1:5000/ 1:3000 | HRP conjugated Goat Anti-Rabbit IgG /HRP conjugated Goat Anti-Rabbit IgG | iF555-Tyramide/ iF488-Tyramide |
| CD86/iNOS | GB115630/GB11119 | Servicebio/Servicebio | Rabbit/Rabbit | 1:1000/ 1:5000 | HRP conjugated Goat Anti-Rabbit IgG /HRP conjugated Goat Anti-Rabbit IgG | iF555-Tyramide/  iF488-Tyramide |
| CD163/ARG1 | GB15340/  GB11285 | Servicebio/Servicebio | Rabbit/Rabbit | 1:3000/ 1:2000 | HRP conjugated Goat Anti-Rabbit IgG /HRP conjugated Goat Anti-Rabbit IgG | iF555-Tyramide/ iF647-Tyramide |
| NF-κB/CD86 | GB11997/  GB115630 | Servicebio/Servicebio | Rabbit/Rabbit | 1:5000/ 1:3000 | HRP conjugated Goat Anti-Rabbit IgG/ HRP conjugated Goat Anti-Rabbit IgG | iF555-Tyramide/  iF488-Tyramide |
| MCP-1/IL-17A | GB11199/  GB11110-1 | Servicebio/Servicebio | Rabbit/Rabbit | 1:3000/  1:3000 | HRP conjugated Goat Anti-Rabbit IgG/ HRP conjugated Goat Anti-Rabbit IgG | iF555-Tyramide/  iF488-Tyramide |
| MCP-1/IL-1β | GB11199/  GB11113 | Servicebio/Servicebio | Rabbit/Rabbit | 1:3000/  1:3000 | HRP conjugated Goat Anti-Rabbit IgG/ HRP conjugated Goat Anti-Rabbit IgG | iF555-Tyramide/  iF488-Tyramide |

The antibodies information for fluorescent homologous double labeling staining

**Supplementary Table S5.** Antibodies used in western blotting.

| Antibodies | Catalog | Vender | Source | Dilution |
| --- | --- | --- | --- | --- |
| IKKβ | A0714 | ABclonal | Rabbit | 1:1000 |
| P50 | A6667 | ABclonal | Rabbit | 1:1000 |
| p-P65 | AP-124 | ABclonal | Rabbit | 1:5000 |
| P65 | 4767T | Cell Signaling | Rabbit | 1:1000 |
| CD86 | ER1906-01 | Huabio | Rabbit | 1:1000 |
| iNOS | 18985-1-AP | Proteintech | Rabbit | 1:2000 |
| CD163 | A26411PM | ABclonal | Rabbit | 1:1000 |
| ARG1 | sc-166920 | Santa | Rabbit | 1:2000 |
| β-actin | 66009-1-g | Proteintech | Mouse | 1:8000 |
| HRP conjugated goat anti-rabbit lgG antibody | CW0103S | CWBIO | Goat | 1:5000 |
| HRP conjugated goat anti-mouse lgG antibody | CW0102S | CWBIO | Goat | 1:5000 |

**Supplementary Table S6. The top 10 upregulated and downregulated DEPs.**

| Protein ID | Symbol | CON | KET | Log2FC | *P* value |
| --- | --- | --- | --- | --- | --- |
| ncbi_541135 | KIF15 | 47.978 | 535.431 | 3.48 | 0.029 |
| ncbi_528582 | KYAT1 | 55.839 | 249.55 | 2.16 | 0.023 |
| ncbi_101903252 | LOC101903252 | 208.951 | 581.276 | 1.476 | 0.033 |
| ncbi_540089 | ANAPC16 | 41.552 | 94.542 | 1.186 | 0.001 |
| ncbi_513301 | PIK3C2G | 21.259 | 44.582 | 1.068 | 0.03 |
| ncbi_281496 | SOD2 | 6580.384 | 13092.681 | 0.993 | 0.009 |
| ncbi_615323 | GRP | 27.895 | 55.343 | 0.988 | 0.022 |
| ncbi_525071 | MTFP1 | 35.515 | 69.98 | 0.979 | 0.021 |
| ncbi_525428 | E2F4 | 13.616 | 24.641 | 0.856 | 0.043 |
| ncbi_516001 | TAF5 | 16.374 | 29.503 | 0.849 | 0.023 |
| ncbi_539836 | ZNF689 | 30 | 10.72 | -1.485 | 0.02 |
| ncbi_615334 | DAO | 339.003 | 140.504 | -1.271 | 0.037 |
| ncbi_508709 | WDR55 | 194.405 | 82.894 | -1.23 | 0.019 |
| ncbi_280981 | PLIN2 | 427.84 | 195.778 | -1.128 | 0.004 |
| ncbi_539889 | INPP5F | 35.283 | 16.204 | -1.123 | 0.03 |
| ncbi_504961 | MPDU1 | 524.551 | 270.961 | -0.953 | 0.025 |
| ncbi_537804 | GRIN2B | 823.043 | 440.134 | -0.903 | 0.01 |
| ncbi_515013 | SCRN2 | 220.638 | 122.311 | -0.851 | 0.041 |
| ncbi_534286 | ALAS1 | 72.826 | 40.52 | -0.846 | 0.04 |
| ncbi_785769 | SPATA13 | 31.995 | 17.954 | -0.834 | 0.002 |

**Supplementary Table S7. The significantly enriched TOP 10 GO terms by GSEA.**

| GO  ID | Classification | Description | Protein number | Core protein number | NES | Pvalue |
| --- | --- | --- | --- | --- | --- | --- |
| GO:0072376 | Biological process | Protein activation cascade | 54 | 39 | 2.592 | < 0.001 |
| GO:2000257 |  | Regulation of protein activation cascade | 29 | 21 | 2.458 | < 0.001 |
| GO:0030198 |  | Extracellular matrix organization | 152 | 81 | 2.443 | < 0.001 |
| GO:0006956 |  | Complement activation | 36 | 24 | 2.429 | < 0.001 |
| GO:0050819 |  | Negative regulation of coagulation | 30 | 22 | 2.420 | < 0.001 |
| GO:0002673 |  | Regulation of acute inflammatory response | 42 | 28 | 2.396 | < 0.001 |
| GO:0003012 |  | Muscle system process | 216 | 99 | 2.39 | < 0.001 |
| GO:0048741 |  | Skeletal muscle fiber development | 21 | 17 | 2.074 | < 0.001 |
| GO:0007599 |  | Hemostasis | 179 | 102 | 2.072 | < 0.001 |
| GO:0014904 |  | Myotube cell development | 22 | 17 | 2.068 | < 0.001 |
| GO:0030016 | Cellular component | Myofibril | 152 | 95 | 2.739 | < 0.001 |
| GO:0031012 |  | Extracellular matrix | 119 | 77 | 2.671 | < 0.001 |
| GO:0043292 |  | Contractile fiber | 158 | 97 | 2.662 | < 0.001 |
| GO:0062023 |  | Collagen-containing extracellular matrix | 67 | 49 | 2.655 | < 0.001 |
| GO:0005604 |  | Basement membrane | 49 | 37 | 2.584 | < 0.001 |
| GO:0030017 |  | Sarcomere | 133 | 82 | 2.578 | < 0.001 |
| GO:0071821 |  | FANCM-MHF complex | 23 | 18 | 2.571 | < 0.001 |
| GO:0032432 |  | Actin filament bundle | 62 | 32 | 2.461 | < 0.001 |
| GO:0042641 |  | Actomyosin | 62 | 38 | 2.429 | < 0.001 |
| GO:0043209 |  | Myelin sheath | 152 | 89 | 2.393 | < 0.001 |
| GO:0008307 | Molecular function | Structural constituent of muscle | 28 | 22 | 2.555 | < 0.001 |
| GO:0005201 |  | Extracellular matrix structural constituent | 29 | 23 | 2.484 | < 0.001 |
| GO:0004866 |  | Endopeptidase inhibitor activity | 81 | 49 | 2.469 | < 0.001 |
| GO:0030414 |  | Peptidase inhibitor activity | 84 | 50 | 2.452 | < 0.001 |
| GO:0005178 |  | Integrin binding | 73 | 37 | 2.032 | < 0.001 |
| GO:0043236 |  | Laminin binding | 21 | 13 | 2.019 | < 0.001 |
| GO:0004857 |  | Enzyme inhibitor activity | 204 | 89 | 2.00 | < 0.001 |
| GO:0004129 |  | Cytochrome-c oxidase activity | 28 | 13 | 1.998 | < 0.001 |
| GO:0008201 |  | Heparin binding | 72 | 36 | 1.995 | < 0.001 |
| GO:0016676 |  | Oxidoreductase activity, acting on a heme group of donors, oxygen as acceptor | 28 | 13 | 1.979 | < 0.001 |

**Supplementary Table S8. The distributed of level 3 pathway by KEGG enrichment analysis.**

| Level2 | Number | Percent |
| --- | --- | --- |
| Immune system | 14 | 10.53% |
| Signal transduction | 12 | 9.02% |
| Endocrine system | 10 | 7.52% |
| Neurodegenerative disease | 7 | 5.26% |
| Nervous system | 7 | 5.26% |
| Cancer: overview | 7 | 5.26% |
| Cardiovascular disease | 6 | 4.51% |
| Infectious disease: bacterial | 6 | 4.51% |
| Lipid metabolism | 5 | 3.76% |
| Infectious disease: viral | 5 | 3.76% |
| Cellular community - eukaryotes | 4 | 3.01% |
| Carbohydrate metabolism | 4 | 3.01% |
| Digestive system | 4 | 3.01% |
| Infectious disease: parasitic | 4 | 3.01% |
| Circulatory system | 3 | 2.26% |
| Immune disease | 3 | 2.26% |
| Cell growth and death | 3 | 2.26% |
| Transport and catabolism | 3 | 2.26% |
| Global and overview maps | 3 | 2.26% |
| Endocrine and metabolic disease | 3 | 2.26% |
| Substance dependence | 3 | 2.26% |
| Metabolism of cofactors and vitamins | 2 | 1.50% |
| Environmental adaptation | 2 | 1.50% |
| Development and regeneration | 2 | 1.50% |
| Excretory system | 2 | 1.50% |
| Replication and repair | 2 | 1.50% |
| Xenobiotics biodegradation and metabolism | 2 | 1.50% |
| Energy metabolism | 1 | 0.75% |
| Signaling molecules and interaction | 1 | 0.75% |
| Cell motility | 1 | 0.75% |
| Cancer: specific types | 1 | 0.75% |
| Folding, sorting and degradation | 1 | 0.75% |

**Supplementary Table S9. The significantly enriched KEGG pathways by GSEA.**

| Pathway  ID | Description | Level 2 | Protein number | Core protein number | NES | Pvalue |
| --- | --- | --- | --- | --- | --- | --- |
| KO04022 | cGMP-PKG signaling pathway | Signal transduction | 77 | 37 | 2.159 | <0.001 |
| KO04024 | cAMP signaling pathway |  | 73 | 29 | 2.025 | <0.001 |
| KO04020 | Calcium signaling pathway |  | 75 | 32 | 2.011 | <0.001 |
| KO04151 | PI3K-Akt signaling pathway |  | 155 | 62 | 1.893 | <0.001 |
| KO04010 | MAPK signaling pathway |  | 122 | 57 | 1.843 | <0.001 |
| KO04066 | HIF-1 signaling pathway |  | 70 | 27 | 1.772 | <0.001 |
| KO04014 | Ras signaling pathway |  | 93 | 26 | 1.688 | <0.001 |
| KO04064 | NF-κB signaling pathway |  | 48 | 9 | 1.526 | 0.017 |
| KO04310 | Wnt signaling pathway |  | 51 | 17 | 1.472 | 0.032 |
| KO04371 | Apelin signaling pathway |  | 69 | 36 | 1.47 | 0.026 |
| KO04072 | Phospholipase D signaling pathway |  | 74 | 41 | 1.433 | 0.044 |
| KO04015 | Rap1 signaling pathway |  | 99 | 44 | 1.394 | 0.023 |
| KO04610 | Complement and coagulation cascades | Immune system | 56 | 42 | 2.67 | <0.001 |
| KO04613 | Neutrophil extracellular trap formation |  | 127 | 58 | 2.362 | <0.001 |
| KO04611 | Platelet activation |  | 80 | 47 | 2.043 | <0.001 |
| KO04666 | Fc gamma R-mediated phagocytosis |  | 70 | 32 | 1.856 | <0.001 |
| KO04062 | Chemokine signaling pathway |  | 88 | 39 | 1.783 | 0.001 |
| KO04640 | Hematopoietic cell lineage |  | 34 | 17 | 1.771 | 0.003 |
| KO04670 | Leukocyte transendothelial migration |  | 74 | 38 | 1.678 | 0.001 |
| KO04621 | NOD-like receptor signaling pathway |  | 102 | 30 | 1.616 | 0.001 |
| KO04662 | B cell receptor signaling pathway |  | 51 | 14 | 1.601 | 0.018 |
| KO04625 | C-type lectin receptor signaling pathway |  | 57 | 20 | 1.582 | 0.01 |
| KO04660 | T cell receptor signaling pathway |  | 54 | 29 | 1.569 | 0.006 |
| KO04658 | Th1 and Th2 cell differentiation |  | 40 | 18 | 1.532 | 0.019 |
| KO04620 | Toll-like receptor signaling pathway |  | 44 | 10 | 1.482 | 0.031 |
| KO04664 | Fc epsilon RI signaling pathway |  | 44 | 18 | 1.473 | 0.038 |
| KO04921 | Oxytocin signaling pathway | Endocrine system | 71 | 44 | 2.078 | <0.001 |
| KO04912 | GnRH signaling pathway |  | 46 | 26 | 1.811 | 0.001 |
| KO04924 | Renin secretion |  | 27 | 12 | 1.728 | 0.005 |
| KO04926 | Relaxin signaling pathway |  | 57 | 19 | 1.682 | 0.007 |
| KO04922 | Glucagon signaling pathway |  | 68 | 39 | 1.604 | 0.007 |
| KO04910 | Insulin signaling pathway |  | 90 | 34 | 1.519 | 0.007 |
| KO04916 | Melanogenesis |  | 33 | 12 | 1.483 | 0.039 |
| KO04935 | Growth hormone synthesis,  secretion and action |  | 62 | 28 | 1.464 | 0.021 |
| KO04915 | Estrogen signaling pathway |  | 58 | 29 | 1.463 | 0.02 |
| KO03320 | PPAR signaling pathway |  | 62 | 15 | -1.517 | 0.017 |

**Supplementary Table S10.** The cytokine alteration in liver tissue between KET group and CON group.

| Protein  ID | AveExp  CON | AveExp KET | Log_2_  (Foldchange) | *P*  Value | Fold change | Regulation | Entrez ID | Uniprot ID | Threshold |
| --- | --- | --- | --- | --- | --- | --- | --- | --- | --- |
| IL-21 | 6.712 | 7.579 | 0.866 | 0.013 | 1.823 | up | 378475 | Q76LU5 | TRUE |
| IGF-1 | 12.079 | 13.129 | 1.050 | 0.005 | 2.070 | up | 281239 | P07455 | TRUE |
| MCP-1 | 10.999 | 11.587 | 0.588 | 0.021 | 1.503 | up | 281043 | P28291 | TRUE |
| IL-10 | 11.990 | 13.114 | 1.124 | 0.019 | 2.180 | up | 281246 | P43480 | TRUE |
| LIF | 12.447 | 13.515 | 1.068 | 0.009 | 2.096 | up | 280840 | Q27956 | TRUE |
| IL-2 | 9.193 | 9.895 | 0.702 | 0.029 | 1.627 | up | 280822 | P05016 | TRUE |
| IL-1β | 6.256 | 7.184 | 0.928 | 0.031 | 1.903 | up | 281251 | P09428 | TRUE |
| IL-17A | 6.078 | 6.736 | 0.659 | 0.038 | 1.579 | up | 282863 | Q687Y7 | TRUE |
| αFGF | 10.696 | 11.146 | 0.450 | 0.062 | 1.366 | up | 281160 | P03968 | FALSE |
| MIG | 5.766 | 6.808 | 1.042 | 0.080 | 2.059 | up | 513990 | A9QWP9 | FALSE |
| IFN-γ | 3.550 | 4.247 | 0.696 | 0.118 | 1.621 | up | 281237 | P07353 | FALSE |
| IL-13 | 7.530 | 6.824 | -0.706 | 0.152 | 0.613 | down | 281247 | Q9XSV9 | FALSE |
| IL-15 | 13.027 | 13.243 | 0.216 | 0.188 | 1.161 | up | 281248 | Q28028 | FALSE |
| IFN-β | 9.006 | 9.416 | 0.409 | 0.181 | 1.328 | up | 281845 | P01578 | FALSE |
| ANG-1 | 7.825 | 8.474 | 0.649 | 0.232 | 1.568 | up | 282140 | O18920 | FALSE |
| IFN-α | 7.705 | 7.908 | 0.203 | 0.262 | 1.151 | up | 515951 | P05007 | FALSE |
| RANTES | 10.194 | 9.549 | -0.645 | 0.291 | 0.640 | down | 327712 | O97919 | FALSE |
| IL-1F5 | 2.340 | 3.344 | 1.004 | 0.315 | 2.005 | up | 518514 | Q0VC52 | FALSE |
| IL-4 | 8.297 | 8.569 | 0.272 | 0.325 | 1.207 | up | 280824 | P30367 | FALSE |
| IP-10 | 6.746 | 6.115 | -0.631 | 0.397 | 0.646 | down | 615107 | Q2KIQ8 | FALSE |
| MIP-1β | 6.240 | 6.452 | 0.212 | 0.387 | 1.158 | up | 414347 | Q17QA1 | FALSE |
| GASP-1 | 5.669 | 5.855 | 0.186 | 0.407 | 1.138 | up | NA | A6QQJ9 | FALSE |
| TNFα | 4.647 | 5.498 | 0.851 | 0.438 | 1.804 | up | 280943 | Q06599 | FALSE |
| βFGF | 8.201 | 8.343 | 0.142 | 0.569 | 1.103 | up | 281161 | P03969 | FALSE |
| CD40L | 11.903 | 12.280 | 0.377 | 0.539 | 1.298 | up | 282387 | P51749 | FALSE |
| IL-18 | 12.331 | 12.444 | 0.113 | 0.566 | 1.082 | up | 281249 | Q9TU73 | FALSE |
| IL-1α | 1.214 | 1.540 | 0.326 | 0.697 | 1.253 | up | 281250 | P08831 | FALSE |
| NCAM-1 | 10.074 | 10.193 | 0.119 | 0.790 | 1.086 | up | 281941 | P31836 | FALSE |
| VEGF | 6.027 | 6.077 | 0.051 | 0.782 | 1.036 | up | 281572 | P15691 | FALSE |
| Decorin | 9.465 | 9.557 | 0.092 | 0.812 | 1.066 | up | 280760 | P21793 | FALSE |

FALSE presented not DECs (KET group compared with CON group).

**Supplementary Table S11. GO enrichment terms of DECs.**

| GO ID | Ontology | Description | *P* value | Gene ID | Count | Richfactor |
| --- | --- | --- | --- | --- | --- | --- |
| GO:0005125 | Molecular function | Cytokine activity | <0.001 | IL21/IL10/IL2/IL1B/IL17A | 5 | 101.875 |
| GO:0048018 |  | Receptor ligand activity | <0.001 | IL21/IGF1/IL10/IL2/IL1B/IL17A | 6 | 31.048 |
| GO:0030546 |  | Signaling receptor activator activity | <0.001 | IL21/IGF1/IL10/IL2/IL1B/IL17A | 6 | 30.803 |
| GO:0030545 |  | Signaling receptor regulator activity | <0.001 | IL21/IGF1/IL10/IL2/IL1B/IL17A | 6 | 30.092 |
| GO:0140677 |  | Molecular function activator activity | <0.001 | IL21/IGF1/IL10/IL2/IL1B/IL17A | 6 | 15.648 |
| GO:0005102 |  | Signaling receptor binding | <0.001 | IL21/IGF1/IL10/IL2/IL1B/IL17A | 6 | 14.543 |
| GO:0098772 |  | Molecular function regulator activity | <0.001 | IL21/IGF1/IL10/IL2/IL1B/IL17A | 6 | 9.731 |
| GO:0005126 |  | Cytokine receptor binding | <0.001 | IL21/IL2/IL1B | 3 | 39.918 |
| GO:0070851 |  | Growth factor receptor binding | <0.001 | IL2/IL1B | 2 | 54.333 |
| GO:0008083 |  | Growth factor activity | 0.002 | IGF1/IL2 | 2 | 28.348 |
| GO:0002700 | Biological process | Regulation of production of molecular mediator of immune response | <0.001 | IL21/IL10/IL2/IL1B/IL17A | 5 | 74.72 |
| GO:0002440 |  | Production of molecular mediator of immune response | <0.001 | IL21/IL10/IL2/IL1B/IL17A | 5 | 64.531 |
| GO:0002697 |  | Regulation of immune effector process | <0.001 | IL21/IL10/IL2/IL1B/IL17A | 5 | 39.436 |
| GO:0002702 |  | Positive regulation of production of molecular mediator of immune response | <0.001 | IL21/IL2/IL1B/IL17A | 4 | 78.328 |
| GO:0002252 |  | immune effector process | <0.001 | IL21/IL10/IL2/  IL1B/IL17A | 5 | 21.51 |
| GO:0002699 |  | Positive regulation of immune effector process | <0.001 | IL21/IL2/IL1B/  IL17A | 4 | 43.683 |
| GO:0002376 |  | Immune system process | <0.001 | IL21/CCL2/IL10/LIF/IL2/IL1B/  IL17A | 7 | 8.063 |
| GO:0002682 |  | Regulation of immune system process | <0.001 | IL21/CCL2/IL10/IL2/IL1B/IL17A | 6 | 11.831 |
| GO:0006955 |  | Immune response | <0.001 | IL21/IL10/LIF/IL2/IL1B/IL17A | 6 | 10.263 |
| GO:0010628 |  | Positive regulation of gene expression | <0.001 | IL21/IL10/IL2/IL1B/IL17A | 5 | 11.733 |
| GO:0005615 | Cellular component | Extracellular space | <0.001 | IL21/IGF1/IL10/  IL2/IL1B/IL17A | 6 | 19.798 |

**Supplementary Table S12. KEGG enrichment pathway of DECs.**

| ID | Description | *P* value | Gene ID | Count | Enrich  factor |
| --- | --- | --- | --- | --- | --- |
| bta04060 | Cytokine-cytokine receptor interaction | <0.001 | IL21/CCL2/IL10/LIF/IL2/IL1B/IL17A | 7 | 25.212 |
| bta04659 | Th17 cell differentiation | <0.001 | IL21/IL2/IL1B/IL17A | 4 | 42.842 |
| bta04630 | JAK-STAT signaling pathway | <0.001 | IL21/IL10/LIF/IL2 | 4 | 22.404 |
| bta04061 | Viral protein interaction with cytokine and cytokine receptor | <0.001 | CCL2/IL10/IL2 | 3 | 38.968 |
| bta04657 | IL-17 signaling pathway | <0.001 | CCL2/IL1B/IL17A | 3 | 38.558 |
| bta04625 | C-type lectin receptor signaling pathway | <0.001 | IL10/IL2/IL1B | 3 | 34.557 |
| bta04668 | TNF signaling pathway | <0.001 | CCL2/LIF/IL1B | 3 | 27.336 |
| bta04672 | Intestinal immune network for IgA production | 0.001 | IL10/IL2 | 2 | 43.607 |
| bta04750 | Inflammatory mediator regulation of TRP channels | 0.003 | IGF1/IL1B | 2 | 22.2 |
| bta04660 | T cell receptor signaling pathway | 0.005 | IL10/IL2 | 2 | 19.078 |
| bta04068 | FoxO signaling pathway | 0.005 | IGF1/IL10 | 2 | 18.089 |
| bta04550 | Signaling pathways regulating pluripotency of stem cells | 0.006 | IGF1/LIF | 2 | 16.841 |
| bta04621 | NOD-like receptor signaling pathway | 0.0106 | CCL2/IL1B | 2 | 12.333 |


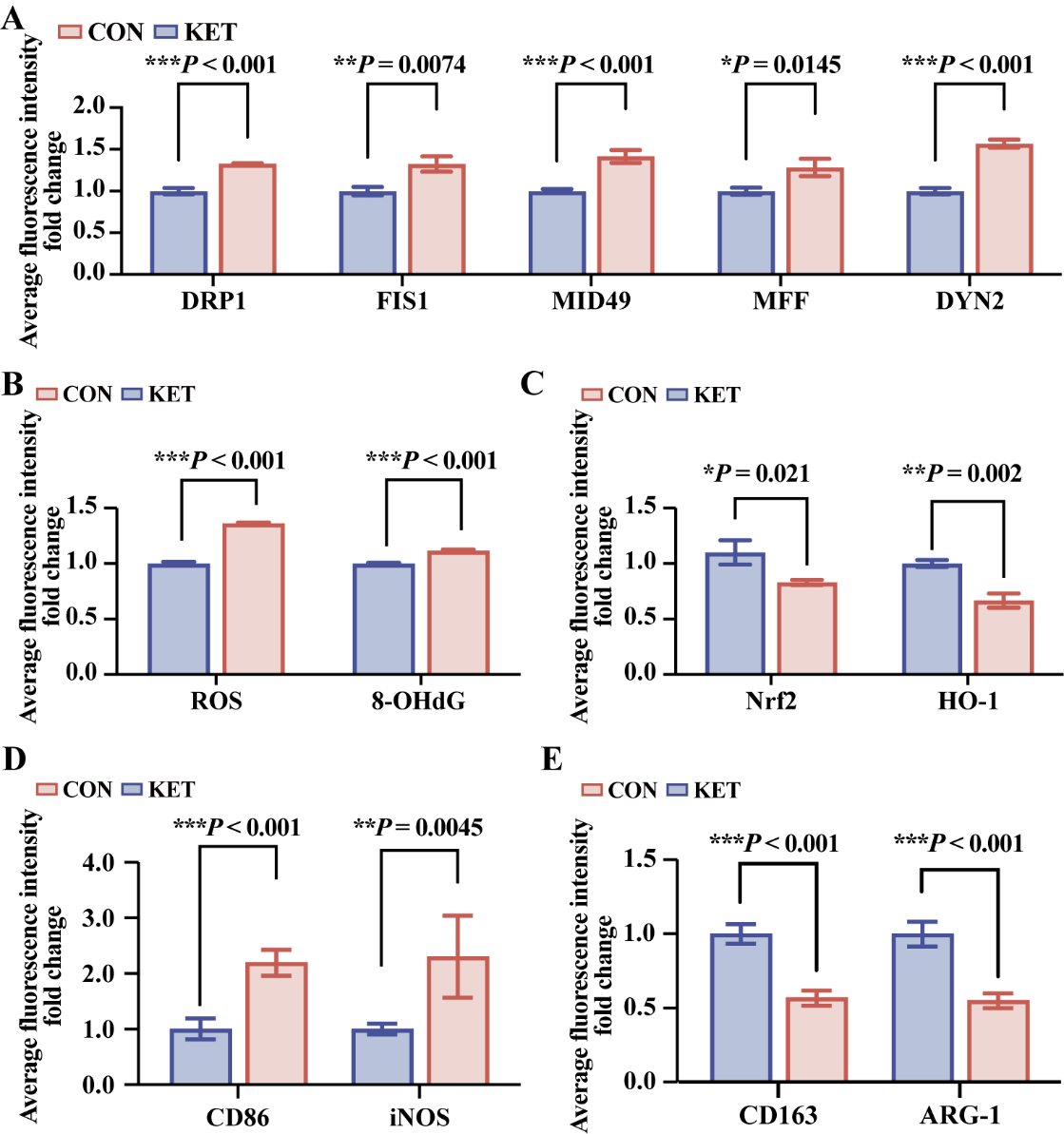


**Fig. S1. Average fluorescence intensity analysis of immunofluorescence sections in the liver in dairy cows with ketosis.** (A) Average fluorescence intensity fold change of DRP1, FIS1, MFF, MID49 and DYN2 (*n* = 5). (B) Average fluorescence intensity fold change of dihydroethidium (DHE) staining and 8-OHdG (*n* = 5). (C) Average fluorescence intensity fold change of Nrf2 and HO-1 (*n* = 5). (D) Average fluorescence intensity fold change of M1 markers (CD86 and iNOS) and M2 markers (CD163 and ARG1) (*n* = 5). Values were expressed as mean with SEM. Statistical analysis was performed using 2-tailed unpaired Student’s t-test. **P* < 0.05, ***P* < 0.01 and ****P* < 0.001.
